# Impact of newborn screening and quality of therapy on the neurological outcome in glutaric aciduria type 1: a meta-analysis

**DOI:** 10.1038/s41436-020-00971-4

**Published:** 2020-09-28

**Authors:** Nikolas Boy, Katharina Mengler, Jana Heringer-Seifert, Georg F. Hoffmann, Sven F. Garbade, Stefan Kölker

**Affiliations:** Division of Child Neurology and Metabolic Medicine, Centre for Child and Adolescent Medicine, University Hospital Heidelberg, Heidelberg, Germany

**Keywords:** glutaric aciduria type 1, glutaric acidemia type 1, newborn screening; outcome; meta-analysis

## Abstract

**Purpose:**

Glutaric aciduria type 1 (GA1), a rare inherited neurometabolic disorder, results in a complex movement disorder (MD) with predominant dystonia if untreated. Implementation into newborn screening (NBS) programs and adherence to recommended therapy are thought to improve the neurological outcome.

**Methods:**

Systematic literature search for articles published from 2000 to 2019 was performed using the PRISMA protocol. Studies reporting on more than one individual identified by NBS were included. We investigated effects of interventional and noninterventional variables on neurological outcome.

**Results:**

Fifteen publications reporting on 647 GA1 patients were included. In the NBS group (*n* = 261 patients), 195 patients remained asymptomatic (74.7%), while 66 patients (25.3%) developed a MD. Compared with the NBS group, a much higher proportion of patients (349/386; 90.4%; *p* < 0.0001) diagnosed after the manifestation of neurologic symptoms had a MD and an abnormal motor development (285/349; 81.7%; *p* < 0.0001). For NBS patients, deviations from the recommended diet increased the risk of insidious onset MD, while delayed start of emergency treatment increased the risk of acute onset MD.

**Conclusions:**

This meta-analysis demonstrates that NBS programs for GA1 have an overall positive effect on the neurological outcome of affected individuals but their success critically depends on the quality of therapy.

## INTRODUCTION

Glutaric aciduria type 1 (GA1, OMIM 231670) is a rare disorder of L-lysine, L-hydroxylysine, and L-tryptophan metabolism caused by inherited deficiency of glutaryl-CoA dehydrogenase (EC 1.3.8.6) resulting in accumulation of glutaryl-CoA and its dicarboxylic derivatives, glutaric acid (GA), 3-hydroxyglutaric acid (3OHGA), glutaconic acid, and glutarylcarnitine in body tissues, especially in the brain. Estimated prevalence ranges from 1:125,000^1^ to 1:250 newborns in genetic high-risk populations.^2–5^ The majority of untreated individuals present with a complex movement disorder (MD) with predominant dystonia mostly between the age of 3 and 36 months due to bilateral striatal damage.^6^ This prognostically relevant event mostly manifests acutely with an acute encephalopathic crisis,^6^ precipitated by catabolism and usually resulting in a severe MD with concomitantly increased morbidity and mortality,^1,6^ or insidiously without clinically apparent crisis, often resulting in a less severe MD compared with the acute manifestation.^1,7–9^ Although GA1 is considered a cerebral organic aciduria, chronic kidney disease has been described recently as the first non-neurologic disease manifestation.^1,10^ Two biochemical subgroups, low (LE) and high excretors (HE), have been arbitrarily defined by the amount of urinary GA excreted, inversely correlating to the residual activity of the deficient enzyme.^11^ The LE phenotype should not be mistaken as attenuated disease variant since both HE and LE individuals share the same high risk of developing MD if untreated.^6,12^ However, a higher frequency of progressive abnormalities, particularly in the white matter, is observed in HE patients, but their clinical relevance is unclear.^13^

Metabolic treatment consisting of a low lysine diet and carnitine supplementation for maintenance treatment (MT) as well as an intermittent emergency treatment (ET) during episodes that are likely to induce catabolism such as febrile infections is recommended by a revised evidence-based guideline.^14^ Most newborn screening (NBS) pilot studies have demonstrated a positive effect of early identification by NBS and neonatal start of treatment on neurologic outcome at variable extent.^1,6,15–20^ However, less beneficial impact of NBS on clinical outcome was observed in a DNA-based program established for the Oji-Cree First Nations in Canada, a known high-risk population with LE phenotype.^21^ In contrast, postsymptomatic treatment is not thought to improve the neurological outcome since striatal damage cannot be reversed.^22^ Therefore, GA1 has been included in a growing number of NBS programs worldwide.^23^ The aim of this meta-analysis of worldwide NBS studies is to evaluate the benefit of NBS programs for individuals with GA1 and to elucidate whether adherence to recommended therapies positively affects the neurologic outcome.

## MATERIALS AND METHODS

### Search strategy

**Fig. 1:**
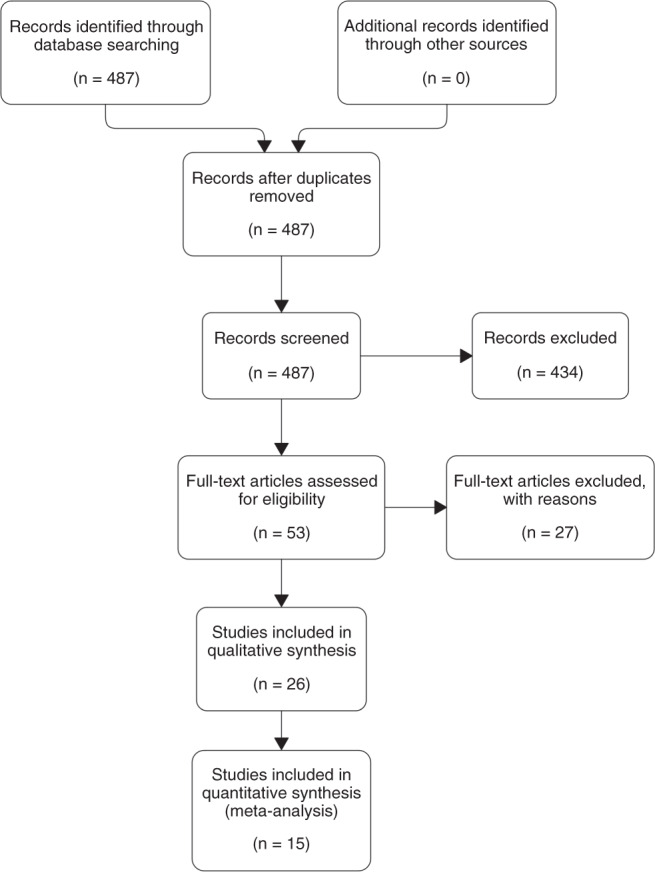
PRISMA flow diagram. The PRISMA flow diagram of systematic literature search demonstrates the flow of information of the meta-analysis. A total of 15 publications reporting on newborn screening (NBS) patients (*n* = 11 for targeted metabolic studies [TMS]) were included into quantitative synthesis after other publications were excluded with reasons. For detailed reasons for exclusion of studies, see Supplementary Tables 2 and  4.

A systematic literature search was performed by the authors and conducted in Medline, Cochrane Library, PubMed, Web of Science, and MeSH databases for reports between 1 January 2000 and 31 December 2019 using the MeSH terms “glutaric aciduria type 1,” “glutaric acidemia type 1,” “glutaryl-CoA dehydrogenase deficiency,” AND “newborn screening,” “treatment,” “outcome.” We used the PRISMA protocol,^24^ which was completed by hand search and contacting authors (Fig. 1).

### Inclusion and exclusion criteria

All abstracts were screened for clinical outcome variables. Only research articles reporting on patients identified by NBS were included. No specific study design was required for inclusion, but reports of single cases were excluded. Studies were only included if collected data fulfilled standard quality criteria, i.e., that the main variables needed for analysis could be extracted. Several studies were excluded with reasons (Fig. 1 and Table S4). In case of multiple publications of the same study population only the most complete and up-to-date publication was included. If a study could not be excluded with certainty, a decision was made after full text evaluation. The study population was defined as patients with confirmed diagnosis of GA1 for whom one or more of the included clinical variables was reported.

### Data extraction

Extracted data contained noninterventional and interventional parameters. Noninterventional parameters comprised gender, biochemical subtype, migrational background, weighted median age at diagnosis, and weighted median age at last visit. Interventional parameters comprised mode of diagnosis, frequency of patients identified by NBS versus patients identified by targeted metabolic studies (TMS, i.e., diagnosis was made after the onset of clinical symptoms), data on clinical phenotype and therapeutic variables (MT and ET, adherence to treatment). Data were extracted from texts, tables, and figures. All extracted variables were transferred to a master MS Excel spread sheet for subsequent analysis (Tables S2, S3) by the authors S.K., N.B., and K.M. Different judgments were solved by agreement. Data were extracted and analyzed separately for patients identified by NBS and TMS as well as for asymptomatic and symptomatic patients (Tables S2, S3). A complete data set of all analyzed variables was not required for inclusion in the quantitative analysis. Due to heterogeneous data quality and amount of reported variables, not all included studies could be used for each analysis. If a specific variable was not reported in a study, the variable was assessed as “not applicable” in the master table resulting in quantitative differences between the total study groups (NBS and TMS group) and subgroup sizes.

### Outcome variables

Patients with a complex MD with predominant dystonia, chorea, or ataxia, or with spastic para- or tetraparesis were defined as “symptomatic,” while asymptomatic individuals had no signs of MD. Onset type of MD was classified as acute onset with an acute encephalopathic crisis, or insidious onset without an apparent crisis event. “Acute encephalopathic crisis” was defined as acute onset of a complex, predominantly dystonic MD after an episode that is likely to precipitate catabolism (e.g., febrile illness) during infancy or childhood in the absence of known alternative causes (e.g., bacterial meningitis). Motor development was assessed as normal or abnormal according to age. The biochemical subtype was assessed using a previous definition.^11^ The variable “migrational background” was defined by at least one parent being born in a foreign-language country different from the patient and included into analysis since language barriers or cultural specifics may have an impact on disease education and treatment adherence.

MT was defined as “according to the guideline” if patients received a low lysine diet with supplementation of a lysine-free, trytophan-reduced, arginine-fortified amino acid mixture (AAS) and carnitine supplementation according to current recommendations.^14^ If another diet (e.g., low protein diet) or no diet was used, MT was defined as “not according to the guideline.” Emergency treatment (ET) was categorized as “according to the guideline” if it was performed according to current recommendations or “not according to the guideline” if it was not performed or started with a delay of more than 24 hours after the onset of alarming symptoms.

This meta-analysis investigated effects of interventional and noninterventional variables on the neurological outcome (frequency and onset type of MD, normal/abnormal motor development, and mortality).

### Statistical analysis

Independent variables used for outcome analysis comprised^1^ adherence to recommended MT,^2^ adherence to recommended ET,^3^ sex,^4^ biochemical subtype, and^5^ migrational background. Two different types of effect sizes were computed. To compare neurological outcome variables between patients identified by NBS and TMS, the Freeman–Tukey double arcsine transformed proportion effect size (PFT) was computed. To compare the effect of adherence with guideline recommendations for MT and ET on a specific neurological outcome variable, the e log based logarithmic risk ratio (RR) was computed. Zero cell counts were replaced by 0.5. A RR above 1 indicates a higher risk and a RR below 1 indicates a reduced risk of neurologic impairment for patients who did not adhere to recommended MT or ET. Effect sizes were further analyzed with a random effect model for meta-analysis and amount of heterogeneity was assessed by DerSimonian–Laird estimator. Results were displayed as forest and funnel plots. Analyses were computed with the statistical package R,^25^ all statistical procedures for computing the meta-analysis were computed with the “metafor” package for R.^26^

## RESULTS

### Study population

Of the 487 identified and screened literature records, 434 records were excluded after abstract evaluation, while 53 full articles were assessed for eligibility, 27 of them having been excluded (single case reports or studies not including NBS patients). The remaining 26 studies were included into qualitative synthesis of which 11 publications were excluded for specific reasons (Fig. 1, Table S4). Finally, a total of 15 publications^1,6,15,16,18–21,27–33^ reporting on more than one NBS patient were included into quantitative synthesis (Fig. 1, Table S2) covering 11 populations in nine countries and two multinational publications (with patients from 37^6^ and 16 countries,^29^ respectively), of which 11 studies^1,6,15,18,20,27–29,31–33^ also reported on patients identified by TMS (Table S3). Overall, reports on 647 GA1 patients were included in the analysis. Total NBS and TMS study populations are summarized in Tables S1–S3.

### Patient groups (Tables S1, S2, S3)

In the NBS group (*n* = 261 patients; female = 124, male = 107, not reported = 30), weighted median age at diagnosis was seven days (range: 4–28 days), and weighted median age at last visit was 43.5 months (range 7–110 months). One hundred seventy-one NBS patients were classified as HE and 53 as LE, while the biochemical phenotype was not reported in 37 individuals. In the TMS group (*n* = 386 patients; female = 139, male = 190, sex not reported = 57) weighted median age at diagnosis was 13.4 months (range 4–144 months), and weighted median age at last visit was 9.3 years (range 2.3–15 years). Biochemical subtypes were reported for 65.8% of TMS patients of whom 167 were classified as HE and 87 as LE.

### Neurological outcome and survival

In the NBS group, age at diagnosis was similar between individuals who remained asymptomatic (weighted median age at diagnosis: seven days, range 7–28 days) compared with those who later on developed neurologic symptoms (weighted median age at diagnosis: 7.07 days, range 7–13 days). Of note, all screened individuals were asymptomatic at time of diagnosis. While the majority of NBS patients (*n* = 195; 74.7%) remained asymptomatic, 66 of them (25.3%) developed a MD with acute (*n* = 39), insidious (*n* = 23) or unreported onset type (*n* = 4). The rate of asymptomatic and symptomatic patients did not differ (*p* = 0.785) between the HE (*n* = 127 asymptomatic, *n* = 44 symptomatic) and LE biochemical subtypes (*n* = 41 asymptomatic, *n* = 12 symptomatic). Motor development was normal in 171 of 195 patients (87.7%) without MD (not reported for *n* = 21), while it was delayed in 48 of 66 of NBS patients with MD (72.7%; not reported for *n* = 18). Nine NBS patients died, the majority of them (*n* = 7) having a severe MD. In addition, two previously asymptomatic patients died, one due to acute renal failure following hemolytic uremic syndrome precipitated by pneumococcal infection and the other patient due to unknown circumstances.

In contrast, most TMS patients (349/386; 90.4%) were symptomatic at time of diagnosis and had a MD with acute (244/349; 69.9%), insidious (79/349; 22.6%), or unreported onset type (26/349; 7.5%). Motor development was delayed in the vast majority of this group (285/349; 81.7%), and 63 (18.1%) symptomatic patients of the TMS group died during the reported study interval. However, a small group of TMS patients (37/386; 9.6%), who were diagnosed due to unspecific clinical signs like muscular hypotonia or macrocephaly (or high-risk screening) but before irreversible neurologic symptoms appeared, had a more favorable outcome. Twenty-two of them (59.5%) had a normal motor development (not reported for *n* = 8), and none of them had died during the study interval. It is noteworthy that patients of this TMS subgroup were diagnosed much earlier (weighted median [range], two [4–144] months) than TMS patients with irreversible neurologic symptoms (weighted median [range], 14 [7–108] months).

### Metabolic maintenance and emergency therapy

The majority of asymptomatic NBS patients (140/195 patients; 71.8%) received MT according to the guideline while 39 patients (20%) received a nonrecommended diet (diet details not reported for 16 individuals). Oral carnitine supplementation was prescribed to all patients. ET according to the guideline was reported for 130/195 (66.6%) of asymptomatic NBS patients, and none of them showed deviations from recommended ET (for 65 asymptomatic NBS patients ET details were not reported). Notably, in NBS patients who developed neurologic symptoms during the documented study interval, the prescribed MT and ET deviated from the recommendations in 48.5% (32/66) and 27.3% (18/66) of symptomatic NBS patients, respectively. Information on ET was not provided in four studies.

In contrast to the NBS group, half of the symptomatic TMS patients (176/349; 50.4%) did not receive recommended MT, and the rate of recommended MT was found to be even lower (11/37; 29.7%) in TMS patients without MD at time of diagnosis. Data on ET were scarce in the TMS group and available only for eight patients.

### Diagnostic impact on the neurological outcome and survival

**Fig. 2:**
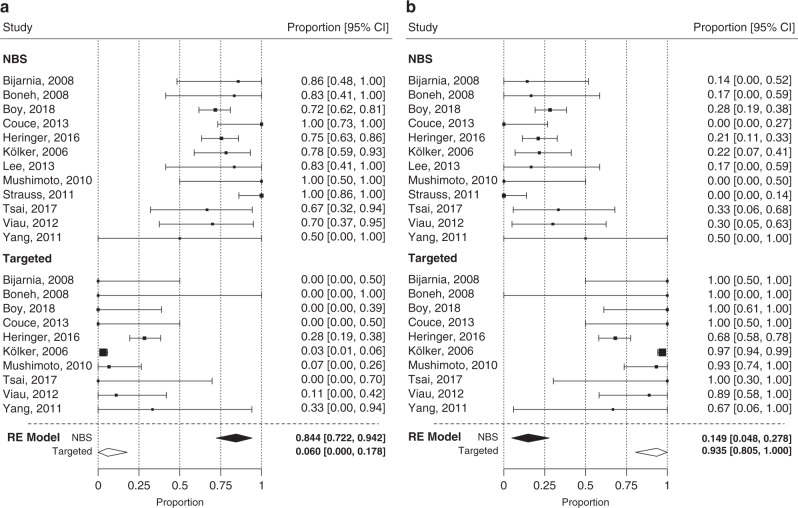
Motor development (normal vs delayed) in patients diagnosed by NBS and Targeted screening. The Random Effect (RE) model measures the mean weighted effect size (indicated by diamonds) and CI (confidence interval). Each square size reflects the study weight. **a** Patients identified by NBS show a significantly higher rate of normal motor development (84.4%). Test of Moderators (coefficient(s) 2): QM(df = 1) = 61.57, *p* < 0.0001, I^2^ (residual heterogeneity/unaccounted variability): 61.38%. The funnel plot showed no remarkable publication bias (for details see Fig. S2A). **b** Patients identified by NBS show a significantly lower rate of delayed motor development (14.9%) than patients identified by TMS. Test of Moderators (coefficient(s) 2): QM(df = 1) = 54.52, *p* < 0.0001, I^2^ (residual heterogeneity/unaccounted variability): 66.05%. The funnel plot showed no remarkable publication bias (for details see Fig. S2B).

**Fig. 3:**
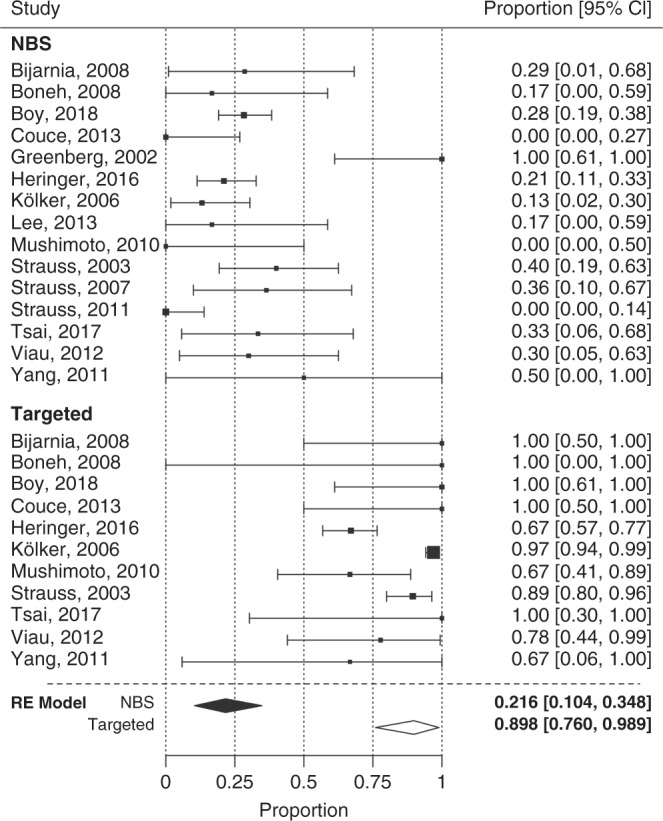
Forest plot for frequency of movement disorders. Patients identified by newborn screening (NBS) show a significantly lower rate of movement disorders than patients identified by targeted metabolic studies (TMS). *CI* confidence interval, *RE* random effect. Test of moderators (coefficient[s] 2): QM(df = 1) = 41.50, *p* < 0.0001. I^2^ (residual heterogeneity/unaccounted variability): 71.93%. Funnel plot showed no remarkable publication bias. (for details see Fig. S3).

**Fig. 4:**
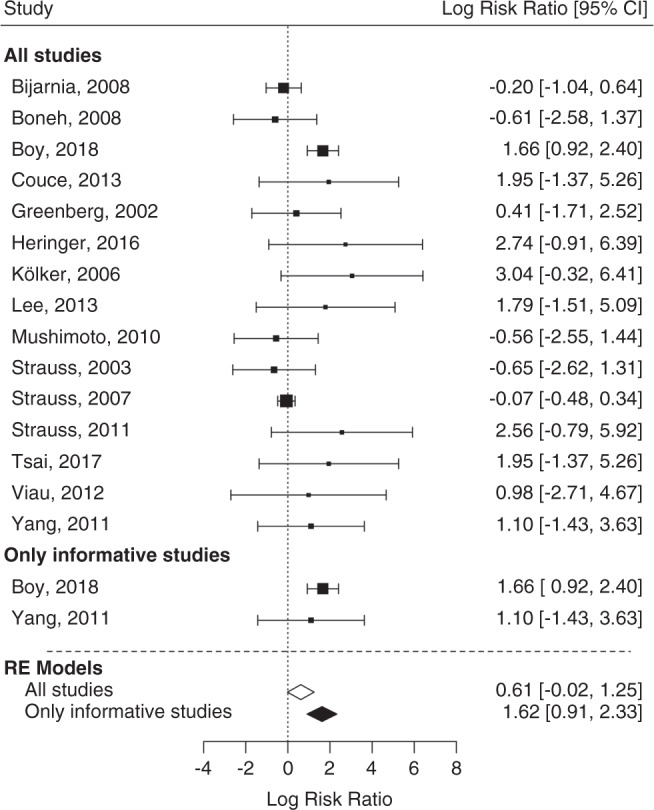
Forest plot for effect of maintenance treatment (MT) adherence and development of insidious onset movement disorder (MD) in patients identified by newborn screening (NBS) including and excluding noninformative studies. In contrast to noninformative studies, informative studies compare two different interventions groups within one study. Patients not following MT recommendations show a trend for increased relative risk (log risk ratio [RR]) for development of an insidious MD compared with patients with MT adherence (*p* = 0.058; log RR 0.61). This effect becomes clearly significant if noninformative studies are excluded (*p* < 0.0001) increasing the RR to almost exp(2) = 7. *CI* confidence interval, *RE* random effect. Including noninformative studies: test of heterogeneity: QM(df14) = 29.1436; model results: *p* = 0.0585. I^2^ (residual heterogeneity/unaccounted variability): 51.28%. Excluding noninformative studies: test for heterogeneity: Q(df = 1) = 0.1765; model results: *p* < 0.0001. I^2^ (residual heterogeneity/unaccounted variability): 0%.

**Fig. 5:**
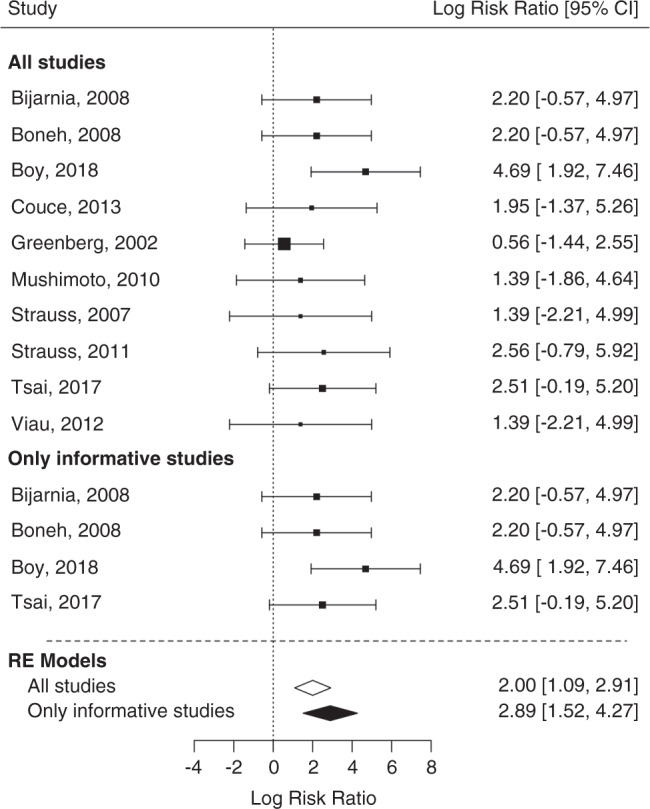
Forest plot for effect of delayed emergency treatment (ET) on development of acute onset movement disorder (MD) in patients identified by newborn screening (NBS) including and excluding noninformative studies. Patients with delayed ET show an increased relative risk (log risk ratio) for development of an acute onset MD compared with patients with adequate ET (*p* < 0.0001). The relative risk increases further if noninformative studies are excluded (*p* < 0.0001). *CI* confidence interval, *RE* random effect. Including noninformative studies: test of heterogeneity: QM(df = 9) = 6.26, *p* = 0.7132; model results: *p* < 0.0001. I^2^ (residual heterogeneity/unaccounted variability): 0%. Excluding noninformative studies: test of heterogeneity QM(df = 3) = 2.1756, *p* = 0.5368; model results: *p* < 0.0001. I^2^ (residual heterogeneity/unaccounted variability): 0%.

To analyze the impact of the age and mode of diagnosis on the neurological outcome and survival, we used a random effect (RE) model measuring mean weighted effect sizes of each study and the 95% confidence interval (CI) and forest plot analysis for the comparison of NBS and TMS patients (Figs. 2–5). Funnel plots showed no publication bias for each analysis (Figs. S2A, B, S3, S4, S5, S7).

### Motor development

Patients identified by NBS showed a significantly higher proportion of normal motor development (mean: 84.4%; 95% CI: 72.2–94.2%) than TMS patients (mean: 6.0%; 95% CI: 0.0–18.8; QM[df = 1] = 61.57; *p* < 0.0001). In accordance, TMS patients showed a higher rate of delayed motor development (mean: 93.5%; 95% CI: 80.5–100.0%) compared with NBS patients (mean: 14.9%; 95% CI: 4.8–27.8%; QM[df = 1] = 54.5; *p* < 0.0001) (Fig. 2a, b).

### Frequency of MD

Patients identified by NBS had an overall lower frequency of MD (mean: 21.6%; 95% CI: 10.4–34.8%) than TMS patients (mean: 89.8%; 95% CI: 76.0–98.9%; QM[df = 1] = 41.5; *p* < 0.0001) (Fig. 3). In analogy, NBS patients developed acute (mean: 11.3%, 95% CI: 3.0–22.3%; QM[df = 1] = 23.78; *p* < 0.0001) (Fig. S1A) and insidious onset MD (mean: 2.1%, 95% CI: 0.0–7.8%; QM[df = 1] = 7.66; *p* = 0.006) (Fig. S1B) less frequently than TMS patients (acute onset MD, mean 63.0%; 95% CI: 45.4–79.3%; insidious onset MD, mean: 17.4%; 95% CI: 7.8–28.9%).

### Survival

Overall mortality of reported individuals with GA1 was low, and mortality rates did not differ between the NBS (mean: 0.2%, 95% CI: 0–5.0%) and TMS groups (mean: 6.4%; 95% CI: 0.3–16.6%; QM[df = 1] = 2.44, *p* = 0.118) (Fig. S6).

### Therapeutic impact on the neurological outcome

Next, we analyzed whether adherence to recommended MT and ET had a beneficial effect on the neurological outcome of screened individuals with GA1.^1^ Since most studies exclusively reported on a single form of MT (according or not according to the guideline) but rarely compared two different forms of MT, separate analyses with inclusion or exclusion of noninformative studies were performed (Figs. 4 and 5). Funnel plot showed no publication bias for each analysis (Figs. S8–S11).

Two studies reporting on a single patient with acute onset MD and nonadherence to recommended MT each were excluded from the analysis on insidious onset MD. We identified nine studies whose patients followed recommended low lysine diet with supplementation of a lysine-free, tryptophan-reduced, and arginine-fortified AAS according to the guideline,^1,6,15,16,19,20,29,32,33^ while patients in another six studies^18,21,27,28,30,31^ were treated with a nonrecommended different diet. Two studies compared different forms of MT.^1,33^

### Nonadherence to MT increases the risk of insidious onset MD

Patients not following MT guideline recommendations showed a trend for increased relative risk (log risk ratio) for insidious onset MD compared with patients with recommended dietary treatment (QM[df = 14] = 29.14; *p* = 0.058; log RR: 0.61; 95% CI: 0.02–1.25). This effect became highly significant (*p* < 0.0001) after exclusion of noninformative studies increasing the RR; however, this analysis was based on two studies, one of them including the so far largest published cohort of screened individuals with GA1^1^ (Fig. 4).

### Nonadherence to ET increases the risk of acute onset MD

Patients with delayed ET had an increased RR (log risk ratio) for acute onset MD compared with patients treated according to recommended ET (QM[df = 9] = 6.26; *p* < 0.0001; log RR: 2, 95% CI: 1.09–2.91). The logarithmized RR increased further after exclusion of noninformative studies (*p* < 0.0001; log RR: 2.89, 95% CI: 1.52–4.27) (Fig. 5).

Effects of other independent variables (Tables S1–S3) such as sex, biochemical subtype, and migrational background had no significant impact on the neurological outcome.

According to^34^ and^35^ detailed evaluation on the risk of bias for each study is summarized in Fig. S12.

## DISCUSSION

Evaluating the best available evidence is essential for assessment of benefits and harms of medical interventions and therefore, meta-analyses have become an important tool for medical research infilling the term “evidence-based medicine.”^36^ Meta-analyses are regularly incorporated to support guideline recommendations and provide the highest level of evidence according to the SIGN^37^ and GRADE^38^ guideline methodology. Structured evaluation of different study designs and populations helps to overcome the limitation of small sample sizes, the overarching challenge of clinical studies on rare diseases. Although meta-analyses usually focus on major common diseases, they become an important tool for research on rare diseases as recently demonstrated for urea cycle disorders.^39^

This meta-analysis of outcomes of individuals with GA1 identified either by NBS or TMS is the most comprehensive synopsis of published data for this rare neurometabolic disorder so far. The main findings are that (1) NBS has an overall beneficial effect on the neurologic outcome of affected individuals, improving motor development and decreasing the frequency of movement disorders; and (2) quality of therapy becomes the major outcome predictor in a screened GA1 population. However, data heterogeneity and low quantity of informative studies comparing different treatment forms hampered some analyses.

### NBS improves the neurological outcome of individuals with GA1

In a growing number of countries, NBS has been introduced over the last 50 years and has improved the outcomes of individuals with inherited metabolic diseases,^23^ becoming a highly effective program of secondary prevention. In GA1, NBS aims at improving the neurologic outcome by preventing irreversible MD and untimely death of symptomatic individuals. In the late 1990s, first NBS pilot studies for GA1 started in Australia, Germany, some US Federal States, and in three genetic high-risk populations (Amish Community, Pennsylvania, USA; Oji-Cree First Nation, Canada; and Irish Travellers, Republic of Ireland). More than 20 years later evidence has increased that early diagnosis and treatment is beneficial.^1,6,15,16,18–20,27,28,30–33^ This meta-analysis unequivocally confirms that patients identified by NBS show a superior neurologic outcome with a higher rate of normal motor development and a lower rate of acute or insidious onset of MD compared with patients identified by TMS. Accordingly, it is a significant progress that a growing number of countries worldwide (e.g., 17/29 European Union member states, according to the International Society for Neonatal Screening) have meanwhile included GA1 in their national NBS programs or NBS pilot studies^23^ and this study will hopefully guide the decision of governments of those countries who have not so far.

### Quality of therapy becomes the major predictor of neurological outcome in a screened population

A previous meta-analysis on GA1 evaluating the effect of metabolic therapy in 155 patients identified by TMS in the prescreening era^22^ concludes that postsymptomatic treatment does not improve the outcome of affected individuals since striatal damage is irreversible. This observation was subsequently confirmed by other studies.^6,7^ As a consequence, there had been considerable uncertainty about the indication and mode of metabolic therapy. It was not before the start of NBS pilot studies that GA1 was shown to be treatable if therapy started before the manifestation of irreversible neurologic symptoms. Since then, treatment strategies for MT and ET have been evaluated, harmonized, and implemented into evidence-based recommendations developed and revised by an international group of experts.^14^ Balanced low lysine diet fortified with lysine-free, tryptophan-reduced, arginine-fortified AAS rather than a low protein diet, which does not calculate the daily lysine intake, was considered the most beneficial^1,6,16,19,20^ and hence is the recommended mode of diet.^14^ In contrast, studies failed to demonstrate a positive effect of low protein diet.^6,28,30^

The largest reported national NBS cohort of GA1 patients (*n* = 87) to date, screened between 1999 and 2016, demonstrated that full adherence to recommendations for both MT and ET was associated with the best neurological outcome and the highest rate of asymptomatic patients (93%),^1^ while 50% of patients with nonrecommended MT and 100% of patients with nonrecommended ET developed a complex MD. These results are supported by the long-term outcome study on neonatally screened Amish patients in Pennsylvania, showing that major improvements in the neurological outcome were achieved following the introduction of ET and low lysine diet.^19^

It is important to note that deviations from recommended treatment had different negative effects on the neurological outcome, with deviations from MT increasing the risk of insidious onset MD, and deviations from ET being highly frequent in individuals with acute onset MD. In other words, quality of therapy becomes the major predictor of neurological outcome in a screened population of GA1.

### Limitations and future challenges in NBS for GA1

The diagnostic quality of glutarylcarnitine screening has been continuously improved using more precise cutoff adjustments^40^ or glutarylcarnitine ratios to other acylcarnitines, resulting in an improved sensitivity but with a significant discrepancy between HE (100%) and LE (84%) patients.^1^ As a consequence, LE patients, who should not be mistaken as having an attenuated disease variant, are still at risk of being missed by NBS programs and hence might still be confronted with poor outcome and reduced life expectancy similar to the prescreening era. To further improve the diagnostic sensitivity for LE patients, a genetic NBS program was started for the Oji-Cree First Nation in Canada, a known high-risk population with LE phenotype.^21^ However, this approach is not suitable for populations with mostly private *GCDH* gene variations like in most countries. LE patients, although exhibiting a lower frequency of extrastriatal abnormalities and intracerebral GA concentrations,^13^ are thought to ultimately share a similar clinical outcome as HE patients with the same a priori risk of developing MD.^1,6^ In line with this, this meta-analysis did not detect an impact of biochemical subtype on outcome.

Although some studies reported on positive NBS effect on survival rates,^6,31^ we could not find a significant difference in reported mortality rates between NBS and TMS patients. This is in contrast to previous studies of the prescreening era,^6,41^ demonstrating about a 50% mortality rate in TMS patients by the age 20–25 years, which might reflect age differences between previous and more recent studies. However, it also highlights that in spite of early identification and start of treatment, current strategies for MT and ET do not reliably prevent the manifestation of severe MD in all screened individuals and untimely death, and notably, more research is needed for development of safer and more effective therapies in GA1.^1^

As NBS and early start of metabolic therapy reduces the frequency of striatal damage, the most important prognostic factor, and allows longer survival, chronic progressive phenotypic changes such as chronic kidney disease^1,10^ and white matter abnormalities become more prominent in screened individuals who have remained asymptomatic until adolescence or adulthood,^13^ necessitating a critical re-evaluation of current strategies for long-term management.

### Study limitations

Meta-analyses have to deal with data heterogeneity, variations in effect sizes and methodological dilemmas and cannot countervail all limitations of the original research. Several studies needed to be excluded for specific reasons. Of the included 15 publications, not all studies could be used for every subanalysis since data quality was heterogeneous and complete data sets could only be retrieved from a minority of studies. Furthermore, included studies often had small sample sizes. For instance, only three studies included into the analysis reported on more than 20 NBS patients,^1,6,29^ and another four studies on more than 10.^19,20,30,31^ While the Freeman–Tukey double arcsine transformed proportion effect sizes that we used for analysis are known to work well for normalizing and variance-stabilizing the sampling distribution of proportions even in small sample sizes, one study^42^ showed problems when back-transforming proportions and sample size ranges are extreme. However, using generalized linear mixed models or random effect models with arcsine square root transformed proportions produced comparable results and does not alter the conclusions.

To avoid unnecessary exclusion of studies and to improve the data quality of studies included into future meta-analyses, the use of patient registries with common data elements and interoperable standardized vocabularies to describe the clinical phenotype, (neuro)development, biochemical and clinical follow-up monitoring, therapy, and other variables are highly recommended.^43^ The collection of complete data sets may provide a powerful tool to increase sample sizes and improve our understanding of long-term outcomes and treatment effects in GA1 and other rare disorders.

### Conclusion

This meta-analysis of outcomes of children with GA1 worldwide identified either by NBS or TMS is a benchmark for care of affected individuals. It demonstrates the overall positive effect of NBS and recommended therapy on neurological outcome. Patients following recommendations for MT have a superior outcome compared with patients following other forms of dietary treatment, while delayed ET increases the risk of acute MD. In contrast, noninterventional parameters have no significant impact on the neurological outcome. Evaluation of therapy, however, is limited by the low quantity of informative studies comparing different treatment forms.

## Supplementary information

Supplementary Material
