# Supplementary material for: Impact of newborn screening and quality of therapy on the neurological outcome in glutaric aciduria type 1: a meta-analysis
Source: Genet Med. 2020 Sep 28;23(1):13–21. doi: 10.1038/s41436-020-00971-4 (PMC7790745; doi:10.1038/s41436-020-00971-4)
Supplement: Supplementary file 1 — Supplementary Material [file 41436_2020_971_MOESM1_ESM.pdf]

## Supplementary Tables

| Study group/ clinical phenotype | Total n | Weighted edian age at diagnosis (days/months) | Weighted median age at last visit (months/years) | Gender | | Biochemical phenotype | | Movement disorder | | | Motor development | | Dea th | Low lysine diet | | Emergency treatment | |
|---|---|---|---|---|---|---|---|---|---|---|---|---|---|---|---|---|---|
| | | | | Female | Male | High excretors (HE) | Low excretors (LE) | Total | Insidious onset | AEC | Normal | Abnormal | | According to guideline | Not according to guideline | According to guideline | Not according to guideline |
| NBS/asymptomatic | 195 | 7 d (4-28) | 35.4 mo (23.3-110.2) | 97 | 79 | 127 | 41 | 0 | 0 | 0 | 171 | 3 | 2 | 140 | 39 | 130 | 0 |
| NBS/symptomatic | 66 | 7.1 d (7-13) | 43.6 mo (7-88.6) | 26 | 28 | 44 | 12 | 66 | 23 | 39 | 0 | 48 | 7 | 29 | 32 | 3 | 18 |
| TMS/asymptomatic | 37 | 13.4 mo (4 -144) | 10.2 years (2.3-10.6) | 17 | 14 | 17 | 4 | 0 | 0 | 0 | 22 | 7 | 0 | 11 | 8 | 8 | 0 |
| TMS/symptomatic | 349 | 13.3 mo (7-108) | 9.3 years (2.6-15) | 122 | 176 | 150 | 83 | 337 | 79 | 244 | 12 | 285 | 63 | 99 | 176 | NA | NA |

**Suppl. Table 1**. Study population of the random effect meta-analysis. In the NBS group (n=261 patients), 169 patients (65%) received recommended therapy; 195 patients remained asymptomatic (74.7%, 95% CI 69.7-98.3) while 66 (25.3%) developed a movement disorder (MD), 39 of whom following acute encephalopathic crisis (EC). In contrast,

63% of TMS patients (n=386) developed an acute encephalopathic crisis ($p<0.0001$). The total number of patients per group (NBS vs TMS) may differ from the numbers of patients included in the sub-groups depending on the reported and specified variables in the study.

*AEC,* encephalopathic crisis; *D,* days; *mo,* months; *NBS,* newborn screening; *HE,* High Excretor*; LE,* Low Excretor; *NA,* not applicable; *TMS,* Targeted metabolic screening

| | | | | | General | | | | | | | | | Clinical phenotype | | | | | | | | MT | | | MT and MD | | | | | | ET | | ET and MD | | | | |
|---|---|---|---|---|---|---|---|---|---|---|---|---|---|---|---|---|---|---|---|---|---|---|---|---|---|---|---|---|---|---|---|---|---|---|---|---|---|
| **N0.** | Country // Subpopulation | Reference | Clinical subtype | ***Year*** | Study Period (year: start end) | **N (NBS patients)** | High Excretors | Low xcretors | Median Age at diagnosis (days) | n (male) | N (female) | n (migrational background) | Median Age at last visit (months) | Movement disorder | Dystonia | Acute encephalopathic crisis | Insidious onset | motor developmen | Delayed motor development | Deceased | Age at death (months) | Low lysine diet | Other diet | No diet | LOW LYSINE NO MD | LOW LYSINE ACUTE MD | LOW LYSINE INSIDIOUS MD | OTHER DIET NO MD | OTHER DIET ACUTE MD | OTHER DIET INSIDIOUS MD | ET according to guideline | no / delayed ET | ET according to guideline NO MD | ET according to guideline WITH ACUTE MD | No/delayed ET NO MD | No/delayed ET ACUTE MD | Metabolic Centre |
| **1** | Amish | Strauss | Asymp | *2003a* | 1989-2002 | 12 | 12 | 0 | 7 | n.a. | n.a. | 0 | 60 | 0 | 0 | 0 | 0 | n.a. | n.a. | 1 | n.a. | 0 | 12 | 0 | 0 | 0 | 0 | 12 | 8 | 0 | 12 | 0 | 12 | 0 | 0 | n.a. | 12 |
| | | | Sympt. | | | 8 | 8 | 0 | 7 | n.a. | n.a. | 0 | 42 | 8 | 8 | 8 | 0 | n.a. | n.a. | 0 | | 0 | 8 | 0 | | | | | | | 8 | 0 | | | | | 8 |
| **2** | Amish | Strauss | Asymp | *2007* | 2003-2005 | 7 | 7 | 0 | 7 | n.a. | n.a. | n.a. | n.a. | 0 | 0 | 0 | 0 | n.a. | n.a. | 0 | | 0 | 7 | 0 | 0 | 0 | 0 | 7 | 1 | 3 | 7 | 0 | 7 | 1 | 0 | 0 | 7 |
| | | | Sympt. | | | 4 | 4 | 0 | 7 | n.a. | n.a. | n.a. | n.a. | 4 | 4 | 1 | 3 | n.a. | n.a. | 0 | | 0 | 4 | 0 | | | | | | | 4 | 0 | | | | | 4 |
| **3** | Amish | Strauss | Asymp | *2011* | 2006-2011 | 12 | 12 | 0 | 4 | 6 | 6 | 12 | 30 | 0 | 0 | 0 | 0 | 12 | 0 | 0 | | 12 | 0 | 0 | 12 | 0 | 0 | 0 | 0 | 0 | 12 | 0 | 12 | 0 | 0 | 0 | 12 |
| | | | Sympt. | | | 0 | 0 | 0 | n.a. | 0 | 0 | 0 | n.a. | 0 | 0 | 0 | 0 | 0 | 0 | 0 | | 0 | 0 | 0 | | | | | | | 0 | 0 | | | | | 0 |
| **4** | Utah, USA | Viau | Asymp | *2012* | n.a. | 7 | 4 | 3 | 10 | 2 | 5 | 5 | 30 | 0 | 0 | 0 | 0 | 7 | 0 | 0 | | 7 | 0 | 0 | 7 | 1 | 2 | 0 | 0 | 0 | 7 | 0 | 7 | 1 | 0 | 0 | 7 |
| | | | Sympt. | | | 3 | 2 | 1 | 12 | 1 | 2 | 1 | 42 | 3 | 3 | 1 | 2 | 0 | 3 | 0 | | 3 | 0 | 0 | | | | | | | 3 | 0 | | | | | 3 |
| **5** | Oji-Cree, Manitoba | Greenberg | Asymp | *2002* | 1998-2001 | 0 | 0 | 0 | n.a. | 0 | 0 | n.a. | n.a. | 0 | 0 | 0 | 0 | n.a. | n.a. | 0 | | 0 | 0 | 0 | 0 | 0 | 0 | 0 | 3 | 1 | 0 | 0 | 0 | 0 | 0 | 3 | 0 |
| | | | Sympt. | | | 4 | 0 | 4 | 9.5 | 1 | 3 | n.a. | 18.5 | 4 | 4 | 3 | 1 | n.a. | n.a. | 1 | 18 | 0 | 4 | 0 | | | | | | | 0 | 4 | | | | | 4 |
| **6** | EIMD-Registry (16 countries) | Heringer | Asymp | *2016* | 2000-2014 | 45 | 18 | 12 | 11 | 21 | 24 | 10 | 26 | 0 | 0 | 0 | 0 | 42 | 1 | 0 | n.a. | 30 | 0 | 0 | 30 | 5 | 1 | 0 | 0 | 0 | n.a. | n.a. | n.a. | n.a. | n.a. | n.a. | 45 |
| | | | Sympt. | | | 12 | 4 | 2 | 7 | 6 | 6 | 7 | 44 | 12 | 11 | 6 | 2 | 1 | 11 | 0 | n.a. | 7 | 0 | 0 | | | | | | | n.a. | n.a. | | | | | 12 |
| **7** | 37 nations, 4 continents | Kölker | Asymp | *2006* | 1999-2004 | 20 | 14 | 6 | 7 | 11 | 9 | 2 | 23.25 | 0 | 0 | 0 | 0 | 18 | 2 | n.a. | | 20 | 0 | 0 | 20 | 2 | 0 | 0 | 1 | 0 | n.a. | n.a. | n.a. | n.a. | n.a. | n.a. | n.a. |
| | | | Sympt. | | | 3 | 2 | 0 | 7 | 2 | 1 | 0 | 41.25 | 3 | 2 | 3 | 0 | 0 | 3 | n.a. | | 2 | 1 | 0 | | | | | | | n.a. | n.a. | | | | | n.a. |
| **8** | Spain | Couce | Asymp | *2013* | 1999-2011 | 6 | 4 | 2 | 16.5 | 2 | 4 | 0 | 56 | 0 | 0 | 0 | 0 | 6 | 0 | 0 | | 6 | 0 | 0 | 6 | 0 | 0 | 0 | 0 | 0 | 6 | 0 | 6 | 0 | 0 | 0 | 6 |
| | | | Sympt. | | | 0 | 0 | 0 | n.a. | 0 | 0 | 0 | n.a. | 0 | 0 | 0 | 0 | 0 | 0 | 0 | | 0 | 0 | 0 | | | | | | | 0 | 0 | | | | | 0 |
| **9** | Germany | Boy | Asymp | *2018* | 1999-2016 | 61 | 42 | 17 | 7 | 28 | 33 | 32 | 110.2 | 0 | 0 | 0 | 0 | 61 | 0 | 1 | 36 | 53 | 8 | 0 | 53 | 9 | 4 | 8 | 2 | 9 | 61 | 0 | 61 | 1 | 0 | 10 | 47 |
| | | | Sympt. | | | 24 | 20 | 4 | 7.5 | 14 | 10 | 15 | 88.6 | 24 | 23 | 11 | 13 | 0 | 24 | 4 | 35.5 | 13 | 11 | 0 | | | | | | | 1 | 10 | | | | | 25 |
| **10** | Australia (New South Wales) | Bijarnia | Asymp | *2008* | 1998-2008 | 5 | n.a. | n.a. | n.a. | 2 | 3 | n.a. | 27 | 0 | 0 | 0 | 0 | 5 | 0 | 0 | | 0 | 4 | 0 | 0 | 0 | 0 | 5 | 1 | 1 | 5 | 0 | 5 | 0 | 0 | 1 | 5 |
| | | | Sympt. | | | 2 | n.a. | n.a. | n.a. | 1 | 1 | n.a. | 11 | 2 | 2 | 1 | 1 | 1 | 1 | 1 | 13 | 0 | 3 | 0 | | | | | | | 1 | 1 | | | | | 2 |
| **11** | Australia (Victoria) | Boneh | Asymp | *2008* | 2001-2007 | 5 | n.a. | n.a. | n.a. | 4 | 1 | n.a. | 36 | 0 | 0 | 0 | 0 | 5 | 0 | 0 | | 0 | 5 | 0 | 0 | 0 | 0 | 5 | 1 | 0 | 5 | 0 | 5 | 0 | 0 | 1 | 5 |
| | | | Sympt. | | | 1 | n.a. | n.a. | n.a. | 1 | 0 | n.a. | 7 | 1 | 1 | 1 | 0 | 0 | 1 | 0 | | 0 | 1 | 0 | | | | | | | 0 | 1 | | | | | 1 |
| **12** | Taiwan | Lee | Asymp | *2013* | 2001-2011 | 5 | 4 | 1 | 12 | 1 | 4 | n.a. | 52 | 0 | 0 | 0 | 0 | 5 | 0 | 0 | | 5 | 0 | 0 | 5 | 1 | 0 | 0 | 0 | 0 | 5 | 0 | n.a. | n.a. | n.a. | n.a. | 4 |
| | | | Sympt. | | | 1 | 1 | 0 | 13 | 0 | 1 | n.a. | 48 | 1 | 1 | 1 | 0 | 0 | 1 | 0 | | 1 | 0 | 0 | | | | | | | 1 | 0 | | | | | 2 |
| **13** | China (Zhejijang) | Yang | Asymp | *2011* | 2008-2010 | 1 | 1 | 0 | 7 | 1 | 0 | n.a. | n.a. | 0 | 0 | 0 | 0 | 1 | 0 | 0 | | 1 | 0 | 0 | 1 | 0 | 0 | 0 | 0 | 1 | 1 | 0 | n.a. | n.a. | n.a. | n.a. | 1 |
| | | | Sympt. | | | 1 | 1 | 0 | 7 | 1 | 0 | n.a. | n.a. | 1 | 1 | 0 | 1 | 0 | 1 | 0 | | 0 | 0 | 0 | | | | | | | 0 | 1 | | | | | 0 |
| **14** | Japan | Mushimoto | Asymp | *2010* | n.a. | 3 | 3 | 0 | 28 | 0 | 3 | n.a. | 64 | 0 | 0 | 0 | 0 | 3 | 0 | 0 | | 0 | 3 | 0 | 0 | 0 | 0 | 3 | 0 | 0 | 3 | 0 | 3 | 0 | 0 | 0 | 3 |
| | | | Sympt. | | | 0 | 0 | 0 | n.a. | 0 | 0 | n.a. | n.a. | 0 | 0 | 0 | 0 | 0 | 0 | 0 | | 0 | 0 | 0 | | | | | | | 0 | 0 | | | | | 0 |
| **15** | Taiwan | Tsai | Asymp | *2017* | 2001-2015 | 6 | 6 | 0 | 8.5 | 1 | 5 | n.a. | 56.5 | 0 | 0 | 0 | 0 | 6 | 0 | 0 | | 6 | 0 | 0 | 6 | 2 | 0 | 0 | 1 | 0 | 6 | 0 | 6 | 0 | 0 | 3 | 4 |
| | | | Sympt. | | | 3 | 2 | 1 | 9 | 1 | 2 | n.a. | 68 | 3 | 3 | 3 | 0 | 0 | 3 | 1 | 12 | 3 | 0 | 0 | | | | | | | 0 | 3 | | | | | 5 |

**Suppl. Table 2.** Master data sheet of all patients identified by NBS reported in the 15 publications included into quantitative analysis. Symptomatic patients were defined as having an acute or insidious onset movement disorder (MD), while patients with no MD were asymptomatic. All patients received oral carnitine supplementation (data not shown).

*Asymp,* asymptomatic; *E-IMD,* European Registry for Intoxication Type Metabolic Diseases; *ET,* emergency treatment; *N.A.,* not applicable; *NBS,* newborn screening; *MD,* movement disorder; *MT,* maintenance treatment; *Symp*, symptomatic

**Comments on selected studies in Suppl. Table 2:**

Strauss et al. 2003: Biochemical subtype was not reported in the study, but all Amish patients show HE phenotype, and all study patients were included into the HE group.

Strauss et al. 2007: Only the 11 patients identified 2003-2005 of this study cohort were included, since the other patients have been also reported in Strauss et al. 2003 and Strauss et al. 2011.

Viau et al. 2012: Patient p4 who was reported to have hypotonia, broad gait and putamen hyperintensity was assessed as ‘symptomatic’.

Greenberg et al. 2002: ET was assessed as delayed in all patients according to the authors (direct author contact).

Heringer et al. 2016: Detailed patient data were not reported in the publication but could be extracted from the E-IMD database after direct author contact. Only patients with a reported daily lysine intake or patients with no diet were included into MT analysis. Patients with ‘calculated diet’ were excluded since exact classification according to the MT guideline recommendations was not possible.

Kölker et al. 2006: ET was not included in the data set of the study.

Boy et al. 2018: Two patients with acute onset of MD after minor head trauma and subdural hemorraghe were excluded. Patients with fine motor deficits (but no signs of MD) were assessed as ‘asymptomatic’. Patients with insidious onset MD (n=11) are not listed in the ET column of the table (resulting in a total of 72 patients with possible ET assessment).

Bijarnia et al. 2008: One patient with influenza infection and mild dyskinesia was reported to be completey resolved and therefore was assessed as ‘asymptomatic’.

Lee et al. 2013: One patient with seizures and normalized pallidal hyperintensity and another patient with focal seizures (both showing no signs of MD) were classified as ‘asymptomatic’.

Yang et al. 2011: One symptomatic patient with therapeutic non-compliance was assessed as insidious onset MD (no acute crisis event reported).

Mushimoto et al. 2010: Details on dietary treament could be extracted after direct author contact (reporting on low protein diet in all patients).

Tsai et al. 2017: Two patients with nystagmus (but no signs of MD) were classified as ‘asymptomatic’.

| Study No. | Country Subpopulation | Reference | Clinical phneotype | | General | | | | | | | | | Clinical phenotype | | | | | | | | Therapy | | | | | | | |
|---|---|---|---|---|---|---|---|---|---|---|---|---|---|---|---|---|---|---|---|---|---|---|---|---|---|---|---|---|---|
| | | | | ***Year*** | Study Period (year: start end) | **N total (TMS patients)** | High Excretors | Low Excretors | Median Age at diagnosis (months) | n (male) | n (female) | n (migration background) | Median age at last visit (years) | Movement disorder | Dystonia | Acute encephalopathic crisis | Insidious onset | Normal motor development | Delayed motor development | Deceased | Median Age at death (years) | Low lysine diet | other diet | no diet | Carnitin | no Carnitin | Emergency Tx | no / delayed emeregncy treatment | Metabolic Centre |
| 1 | USA | Strauss | Asympt. | *2003a* | 1989-2002 | 6 | n.a. | n.a. | 144 | n.a. | n.a. | 0 | 10 | 0 | 0 | 0 | 0 | n.a. | n.a. | 0 | | 0 | 6 | 0 | 6 | 0 | 6 | 0 | 6 |
| | | | Sympt. | | | 51 | n.a. | n.a. | 108 | n.a. | n.a. | 0 | 7.5 | 51 | 50 | 28 | 22 | n.a. | n.a. | 7 | 9 | 0 | 51 | 0 | 51 | 0 | 51 | 0 | 51 |
| 2 | Utah, USA | Viau | Asympt. | *2012* | n.a. | 0 | 0 | 0 | n.a. | 0 | 0 | 0 | n.a. | 0 | 0 | 0 | 0 | 0 | 0 | 0 | | 0 | 0 | 0 | 0 | 0 | 0 | 0 | 0 |
| | | | Sympt. | | | 9 | 4 | 3 | 8 | 4 | 5 | 1 | 12 | 7 | 7 | n.a. | n.a. | 1 | 8 | 0 | | 5 | 0 | 4 | 7 | 2 | n.a. | n.a. | 9 |
| 3 | EIMD-registry | Heringer | Asympt. | *2016* | 1975-2014 | 29 | 15 | 4 | 19.6 | 13 | 16 | 10 | 10.6 | 0 | 0 | 0 | 0 | 20 | 7 | 0 | n.a. | 11 | 0 | 0 | 26 | 3 | n.a | n.a | 27 |
| | | | Sympt. | | | 59 | 15 | 15 | 12.8 | 36 | 23 | 7 | 12.7 | 59 | 52 | 36 | 16 | 5 | 53 | 1 | 8.9 | 19 | 0 | 0 | 52 | 7 | n.a. | n.a. | 57 |
| 4 | 37 nations, 4 continents | Kölker | Asympt. | *2006* | 1999-2004 | 0 | 0 | 0 | n.a. | 0 | 0 | 0 | n.a. | 0 | 0 | 0 | 0 | 0 | 0 | 0 | | 0 | 0 | 0 | 0 | 0 | 0 | 0 | 0 |
| | | | Sympt. | | | 201 | 113 | 58 | 14 | 119 | 82 | 19 | 9.6 | 195 | 162 | 160 | 36 | 6 | 195 | 50 | 6.6 | 70 | 105 | 26 | 171 | 27 | n.a. | n.a. | n.a. |
| 5 | Spain | Couce | Asympt. | *2013* | 1999-2011 | 0 | 0 | 0 | n.a. | 0 | 0 | 0 | n.a. | 0 | 0 | 0 | 0 | 0 | 0 | 0 | | 0 | 0 | 0 | 0 | 0 | 0 | 0 | 0 |
| | | | Sympt. | | | 3 | 1 | 2 | 14 | 3 | 0 | 0 | 12.1 | 3 | 2 | 2 | 1 | 0 | 3 | 0 | | 1 | 2 | 0 | 3 | 0 | 1 | 2 | 3 |
| 6 | Germany | Boy | Asympt. | *2018* | 1999-2016 | 0 | 0 | 0 | | 0 | 0 | 0 | n.a. | 0 | 0 | 0 | 0 | 0 | 0 | 0 | | 0 | 0 | 0 | 0 | 0 | 0 | 0 | 0 |
| | | | Sympt. | | | 4 | 0 | 4 | 35 | 0 | 4 | 3 | 6.25 | 4 | 4 | 4 | 0 | 0 | 4 | 1 | 3.3 | 0 | 0 | 4 | 0 | 4 | 4 | 0 | 3 |
| 7 | Australia (New South Wales) | Bijarnia | Asympt. | *2008* | 1998-2008 | 0 | n.a. | n.a. | n.a. | 0 | 0 | n.a. | n.a. | 0 | 0 | 0 | 0 | 0 | 0 | 0 | | 0 | 0 | 0 | 0 | 0 | 0 | 0 | 0 |
| | | | Sympt. | | | 3 | n.a. | n.a. | 10 | 2 | 1 | n.a. | 9 | 3 | 3 | 1 | 2 | 0 | 3 | 0 | | 0 | 3 | 0 | 3 | 0 | n.a. | n.a. | 3 |
| 8 | Australia (Victoria) | Boneh | Asympt. | *2008* | 2001-2007 | 0 | n.a. | n.a. | n.a. | 0 | 0 | n.a. | n.a. | 0 | 0 | 0 | 0 | 0 | 0 | 0 | | 0 | 0 | 0 | 0 | 0 | 0 | 0 | 0 |
| | | | Sympt. | | | 1 | n.a. | n.a. | 48 | 1 | 0 | n.a. | 7 | 1 | 1 | 0 | 1 | 0 | 1 | 0 | | 0 | 1 | 0 | 1 | 0 | 1 | 0 | 1 |
| 9 | China | Yang | Asympt. | *2011* | 2008-2010 | 1 | 1 | 0 | 4 | 0 | 1 | n.a. | 2.3 | 0 | 0 | 0 | 0 | 1 | 0 | 0 | | 0 | 1 | 0 | 1 | 0 | 1 | 0 | 1 |
| | | | Sympt. | | | 2 | 2 | 0 | 18 | 1 | 1 | n.a. | 2.6 | 2 | 2 | 2 | 0 | 0 | 2 | 0 | | 2 | 0 | 0 | 2 | 0 | 2 | 0 | 2 |
| 10 | Japan | Mushimoto | Asympt. | *2010* | n.a. | 1 | 1 | 0 | 27 | 1 | 0 | n.a. | 6 | 0 | 0 | 0 | 0 | 1 | 0 | 0 | | 0 | 1 | 0 | 1 | 0 | 1 | 0 | 1 |
| | | | Sympt. | | | 14 | 14 | 0 | 7 | 9 | 5 | n.a. | 4.4 | 10 | 10 | 9 | 1 | 0 | 14 | 3 | 4 | 0 | 14 | 0 | 14 | 0 | 14 | 0 | 14 |
| 11 | Taiwan | Tsai | Asympt. | *2017* | 2001-2015 | 0 | 0 | 0 | n.a. | 0 | 0 | 0 | n.a. | 0 | 0 | 0 | 0 | 0 | 0 | 0 | | 0 | 0 | 0 | 0 | 0 | 0 | 0 | 0 |
| | | | Sympt. | | | 2 | 1 | 1 | 89.25 | 1 | 1 | n.a. | 15 | 2 | 2 | 2 | 0 | 0 | 2 | 1 | 7.3 | 2 | 0 | 0 | 2 | 0 | 2 | 0 | 2 |

**Suppl. Table 3.** Master data sheet of all patients identified by targeted metabolic screening (TMS) that are reported in the 11 publications included into quantitative analysis. Symptomatic patients were defined as having an acute or insidious onset movement disorder (MD), while patients with no MD were asymptomatic.

For detailed comments on selected studies see Suppl. Table 2.

*N.A.,* not applicable; *NBS,* newborn screening; *MD,* movement disorder; *TMS,* targeted metabolic screening

| Study | Reason for exclusion (if applicable) |
|---|---|
| 1. Beauchamp et al. Journal of inherited metabolic disease. 2009;32 Suppl 1:S207-13. | Clinical history of four patients has been reported by Boneh et al. 2008 |
| 2. Bijarnia et al. Journal of inherited metabolic disease. 2008;31(4):503-7. | |
| 3. Boneh et al. Molecular genetics and metabolism. 2008;94(3):287-91. | |
| 4. Boy et al. Journal of inherited metabolic disease. 2013;36(3):525-33. | Patients also included in the more up-to-date study cohort of Boy et al. 2018 |
| 5. Boy et al. Orphanet journal of rare diseases. 2015;10:163. | Patients also included in the up-to-date study cohort of Boy et al. 2018 |
| 6. Boy et al. Annals of neurology. 2018;83(5):970-9. | |
| 7. Brown et al. JIMD reports. 2015;18:125-34. | Clinical history of these patients has been reported by Boneh et al. 2008 |
| 8. Couce ML, et al. European journal of paediatric neurology : EJPN : official journal of the European Paediatric Neurology Society. 2013;17(4):383-9. | |
| 9. Greenberg CR, et al. Molecular genetics and metabolism. 2002;75(1):70-8. | |
| 10. Heringer et al. Annals of neurology. 2010;68(5):743-52. | Patients also included in the more up-to-date study cohort of Boy et al. 2018 |
| 11. Heringer et al. Journal of inherited metabolic disease. 2016;39(3):341-53. | |
| 12. Hsieh et al. J Formos Med Assoc. 2008;107(2):139-44. | Clinical history of four patients also reported by Lee et al. 2013 |
| 13. Kolker et al. Molecular genetics and metabolism. 2012;107(1-2):72-80. | Patients also included in the more up-to-date study cohort of Boy et al. 2018 |
| 14. Kolker et al. Pediatric research. 2007;62(3):357-63 | Patients also included in the more up-to-date study cohort of Boy et al. 2018 |
| 15. Kolker et al. Pediatric research. 2006;59(6):840-7. | |
| 16. Kolker et al. Journal of inherited metabolic disease. 2015;38(6):1041-57 | Patients also included in the more up-to-date E-IMD publication of Heringer et al. 2016 |
| 17. Kolker et al. Journal of inherited metabolic disease. 2015;38(6):1059-74. | Patients also included in the more up-to-date E-IMD publication of Heringer et al. 2016 |
| 18. Kyllerman et al. European journal of paediatric neurology : EJPN : official journal of the European Paediatric Neurology Society. 2004;8(3):121-9. | Only patients identifed by targeted screening reported |
| 19. Lee et al. Metab Brain Dis. 2013;28(1):61-7. | |
| 20. Mushimoto et al.Molecular genetics and metabolism. 2011;102(3):343-8. | |
| 21. Strauss et al. Molecular genetics and metabolism. 2011;104(1-2):93-106. | |
| 22. Strauss et al. Brain : a journal of neurology. 2007;130(Pt 7):1905-20. | |
| 23. Strauss et al. American journal of medical genetics Part C, Seminars in medical genetics. 2003;121C(1):38-52. | |
| 24. Tsai et al. J Chin Med Assoc. 2017;80(4):253-61. | |
| 25. Viau K et al. Molecular genetics and metabolism. 2012;106(4):430-8. | |
| 26. Yang L et al.Med Sci Monit. 2011;17(7):PH55-9. | |

**Suppl. Table 4.** List of publications (alphabetic order) included into qualitative synthesis (n=26) and specification of exclusion.

Additional references of excluded publications (n=11) that are not listed in the main text:

(1-11)

1. Beauchamp MH, Boneh A, Anderson V. Cognitive, behavioural and adaptive profiles of children with glutaric aciduria type I detected through newborn screening. Journal of inherited metabolic disease. 2009;32 Suppl 1:S207-13.
2. Boy N, Haege G, Heringer J, Assmann B, Muhlhausen C, Ensenauer R, et al. Low lysine diet in glutaric aciduria type I--effect on anthropometric and biochemical follow-up parameters. Journal of inherited metabolic disease. 2013;36(3):525-33.
3. Boy N, Heringer J, Haege G, Glahn EM, Hoffmann GF, Garbade SF, et al. A cross-sectional controlled developmental study of neuropsychological functions in patients with glutaric aciduria type I. Orphanet journal of rare diseases. 2015;10:163.
4. Brown A, Crowe L, Beauchamp MH, Anderson V, Boneh A. Neurodevelopmental profiles of children with glutaric aciduria type I diagnosed by newborn screening: a follow-up case series. JIMD reports. 2015;18:125-34.
5. Heringer J, Boy SP, Ensenauer R, Assmann B, Zschocke J, Harting I, et al. Use of guidelines improves the neurological outcome in glutaric aciduria type I. Annals of neurology. 2010;68(5):743-52.
6. Hsieh CT, Hwu WL, Huang YT, Huang AC, Wang SF, Hu MH, et al. Early detection of glutaric aciduria type I by newborn screening in Taiwan. J Formos Med Assoc. 2008;107(2):139-44.
7. Kolker S, Boy SP, Heringer J, Muller E, Maier EM, Ensenauer R, et al. Complementary dietary treatment using lysine-free, arginine-fortified amino acid supplements in glutaric aciduria type I - A decade of experience. Molecular genetics and metabolism. 2012;107(1-2):72-80.
8. Kolker S, Garbade SF, Boy N, Maier EM, Meissner T, Muhlhausen C, et al. Decline of acute encephalopathic crises in children with glutaryl-CoA dehydrogenase deficiency identified by newborn screening in Germany. Pediatric research. 2007;62(3):357-63.
9. Kolker S, Garcia-Cazorla A, Valayannopoulos V, Lund AM, Burlina AB, Sykut-Cegielska J, et al. The phenotypic spectrum of organic acidurias and urea cycle disorders. Part 1: the initial presentation. Journal of inherited metabolic disease. 2015;38(6):1041-57.
10. Kolker S, Valayannopoulos V, Burlina AB, Sykut-Cegielska J, Wijburg FA, Teles EL, et al. The phenotypic spectrum of organic acidurias and urea cycle disorders. Part 2: the evolving clinical phenotype. Journal of inherited metabolic disease. 2015;38(6):1059-74.
11. Kyllerman M, Skjeldal O, Christensen E, Hagberg G, Holme E, Lonnquist T, et al. Long-term follow-up, neurological outcome and survival rate in 28 Nordic patients with glutaric aciduria type 1. European journal of paediatric neurology : EJPN : official journal of the European Paediatric Neurology Society. 2004;8(3):121-9.

**Supplementary Figures**

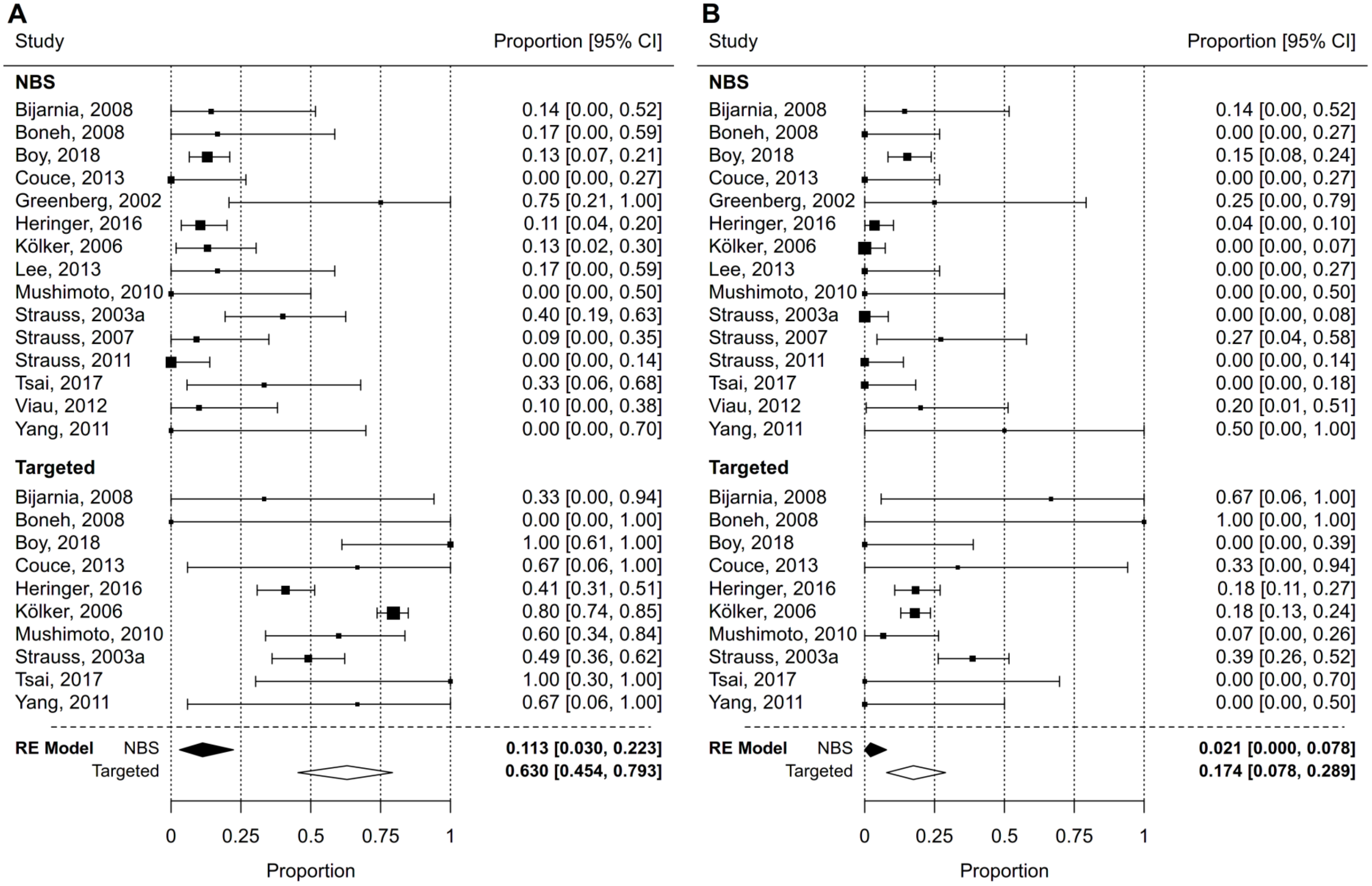
A
Study
Proportion [95% CI]
NBS
Bijarnia, 2008 0.14 [0.00, 0.52]
Boneh, 2008 0.17 [0.00, 0.59]
Boy, 2018 0.13 [0.07, 0.21]
Couce, 2013 0.00 [0.00, 0.27]
Greenberg, 2002 0.75 [0.21, 1.00]
Heringer, 2016 0.11 [0.04, 0.20]
Kölker, 2006 0.13 [0.02, 0.30]
Lee, 2013 0.17 [0.00, 0.59]
Mushimoto, 2010 0.00 [0.00, 0.50]
Strauss, 2003a 0.40 [0.19, 0.63]
Strauss, 2007 0.09 [0.00, 0.35]
Strauss, 2011 0.00 [0.00, 0.14]
Tsai, 2017 0.33 [0.06, 0.68]
Viau, 2012 0.10 [0.00, 0.38]
Yang, 2011 0.00 [0.00, 0.70]
Targeted
Bijarnia, 2008 0.33 [0.00, 0.94]
Boneh, 2008 0.00 [0.00, 1.00]
Boy, 2018 1.00 [0.61, 1.00]
Couce, 2013 0.67 [0.06, 1.00]
Heringer, 2016 0.41 [0.31, 0.51]
Kölker, 2006 0.80 [0.74, 0.85]
Mushimoto, 2010 0.60 [0.34, 0.84]
Strauss, 2003a 0.49 [0.36, 0.62]
Tsai, 2017 1.00 [0.30, 1.00]
Yang, 2011 0.67 [0.06, 1.00]
RE Model NBS 0.113 [0.030, 0.223]
Targeted 0.630 [0.454, 0.793]
0 0.25 0.5 0.75 1
Proportion
B
Study
Proportion [95% CI]
NBS
Bijarnia, 2008 0.14 [0.00, 0.52]
Boneh, 2008 0.00 [0.00, 0.27]
Boy, 2018 0.15 [0.08, 0.24]
Couce, 2013 0.00 [0.00, 0.27]
Greenberg, 2002 0.25 [0.00, 0.79]
Heringer, 2016 0.04 [0.00, 0.10]
Kölker, 2006 0.00 [0.00, 0.07]
Lee, 2013 0.00 [0.00, 0.27]
Mushimoto, 2010 0.00 [0.00, 0.50]
Strauss, 2003a 0.00 [0.00, 0.08]
Strauss, 2007 0.27 [0.04, 0.58]
Strauss, 2011 0.00 [0.00, 0.14]
Tsai, 2017 0.00 [0.00, 0.18]
Viau, 2012 0.20 [0.01, 0.51]
Yang, 2011 0.50 [0.00, 1.00]
Targeted
Bijarnia, 2008 0.67 [0.06, 1.00]
Boneh, 2008 1.00 [0.00, 1.00]
Boy, 2018 0.00 [0.00, 0.39]
Couce, 2013 0.33 [0.00, 0.94]
Heringer, 2016 0.18 [0.11, 0.27]
Kölker, 2006 0.18 [0.13, 0.24]
Mushimoto, 2010 0.07 [0.00, 0.26]
Strauss, 2003a 0.39 [0.26, 0.52]
Tsai, 2017 0.00 [0.00, 0.70]
Yang, 2011 0.00 [0.00, 0.50]
RE Model NBS 0.021 [0.000, 0.078]
Targeted 0.174 [0.078, 0.289]
0 0.25 0.5 0.75 1
Proportion

**Figure S1 A, B. Forest Plot for frequency of acute encephalopathic crisis (A) and insidious onset MD (B)**

Random Effect (RE) model shows that patients identified by NBS show a significantly lower rate of acute encephalopathic crises a as well as significantly lower rate of insidious onset MD than patients identified by TMS.

MD, movement disorder; NBS, newborn screening; RE, random effect; TMS, targeted metabolic studies

Fig 3A:

Test of Moderators (coefficient(s) 2): QM(df=1)=23.78, $p<0.0001$

I^2(residual heterogeneity/unaccounted variability): 70.07%.

Funnel Plot showed no remarkable publication bias (for details see Fig. S4).

Fig 3B:

Random Effect (RE) model shows that

Test of Moderators (coefficient(s) 2): QM(df=1)=7.66, $p=0.0057$

I^2(residual heterogeneity/unaccounted variability): 48.68%.

Funnel Plot showed no remarkable publication bias (for details see Fig. S5).

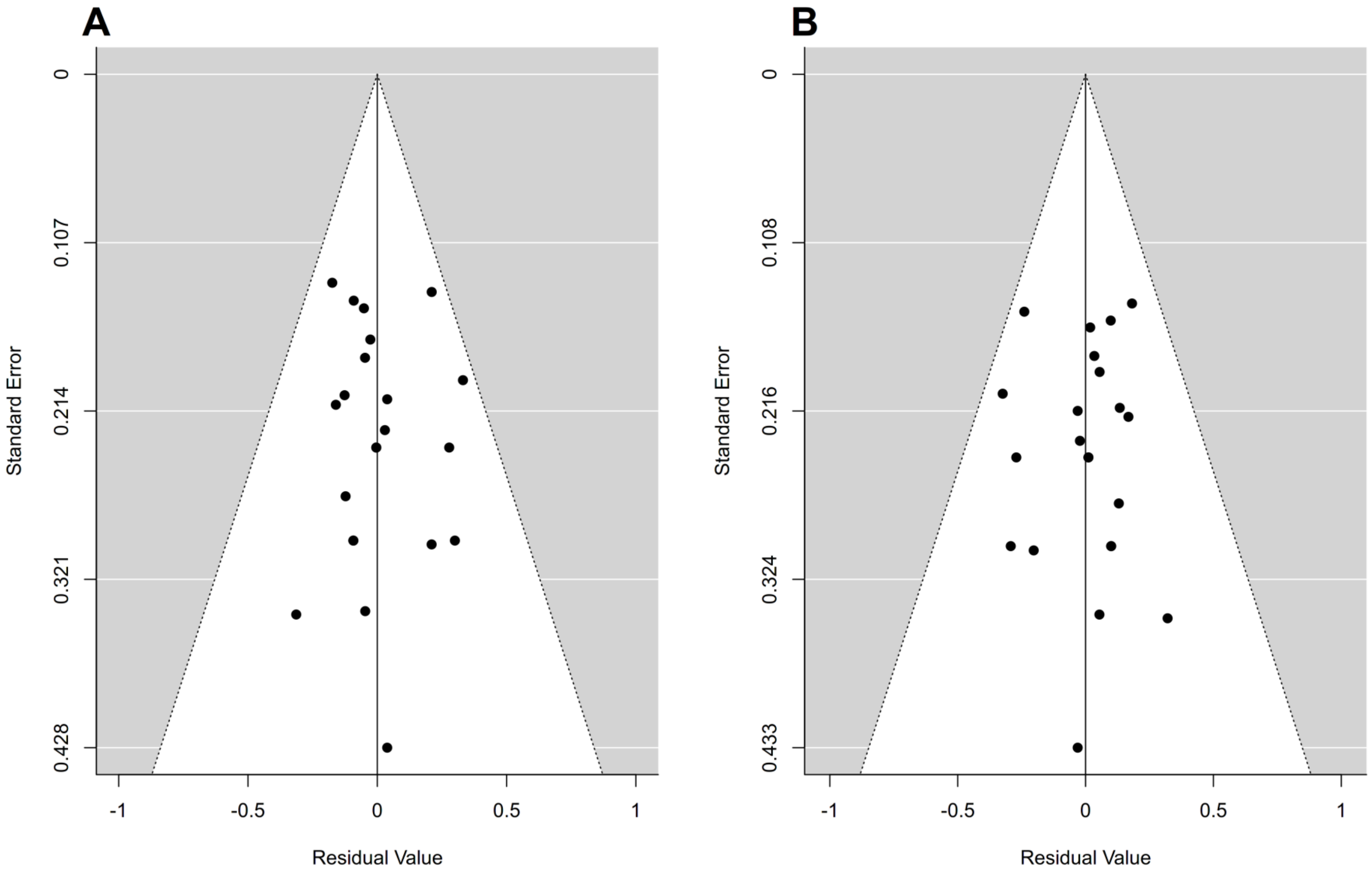


**Figure S2. Funnel Plots for frequency of normal (A) and delayed (B) motor development showing residual value and standard error of included publications.** The analysis showed no remarkable publication bias.

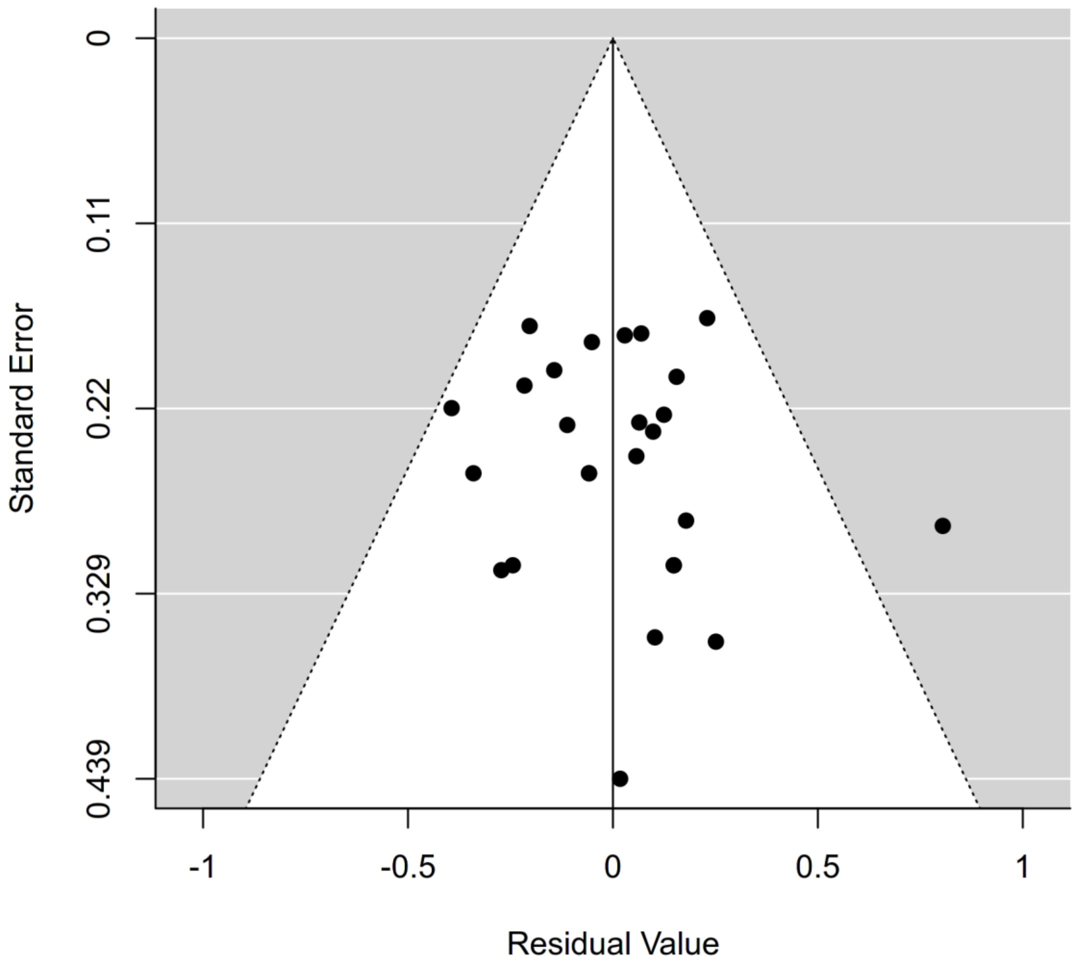


**Figure S3. Funnel-Plot for frequency of movement disorders showing residual value and standard error of included publications.** The analysis showed no remarkable publication bias.

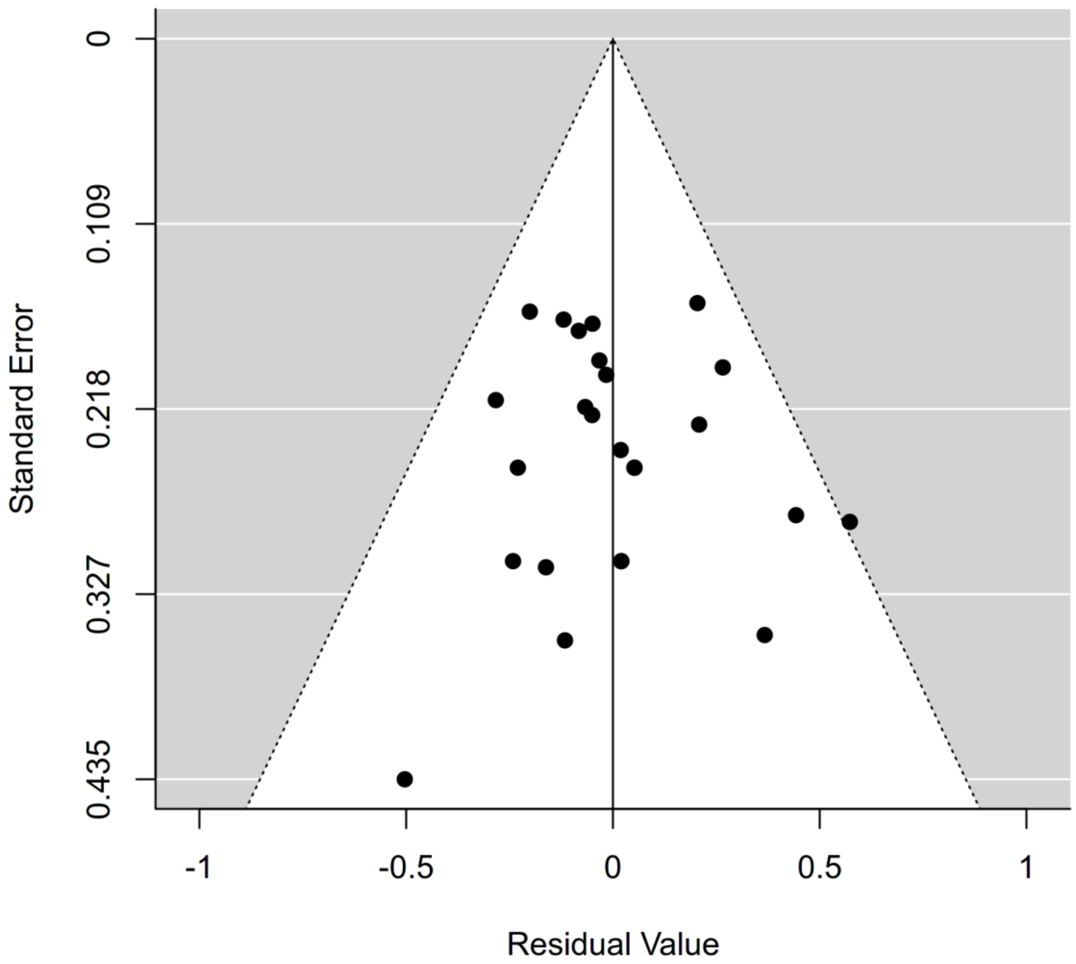


**Figure S4. Funnel-Plot for frequency of acute encephalopathic crisis showing residual value and standard error of included publications**. The analysis showed no remarkable publication bias.

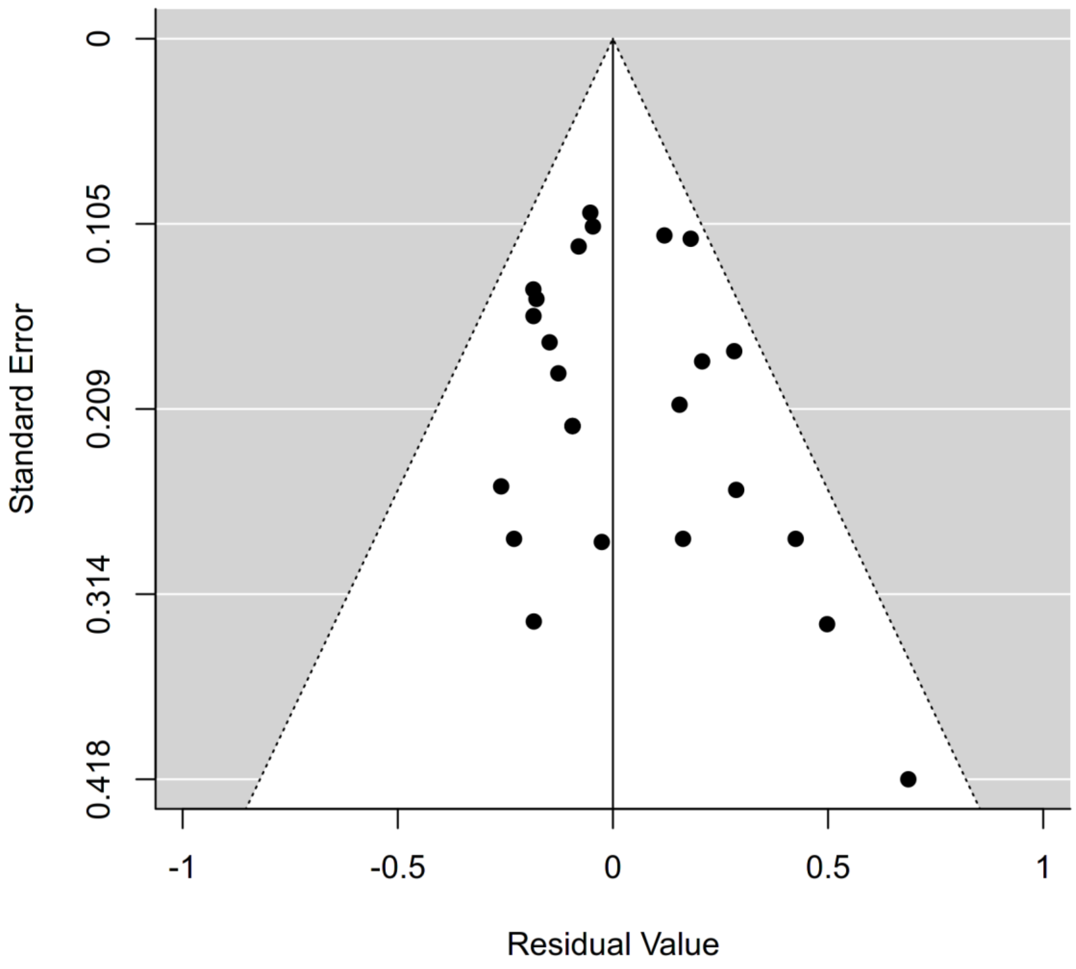


**Figure S5. Funnel-Plot for frequency of insidious onset MD showing residual value and standard error of included publications.** The analysis showed no remarkable publication bias.

*MD,* movement disorder

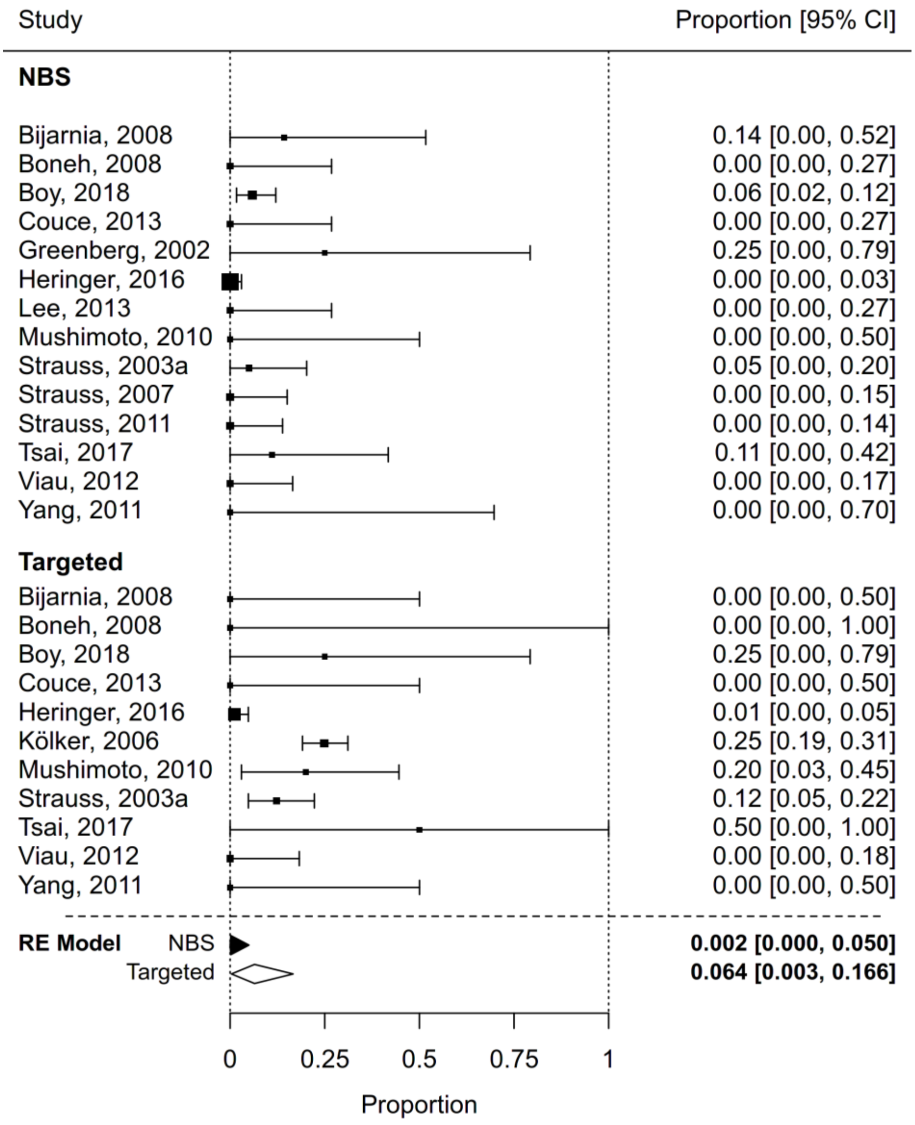


**Figure S6. Forest Plot for frequency of deceased patients.** Patients identified by NBS do not significantly differ from patients identified by TMS.

*NBS,* newborn screening; *TMS,* targeted metabolic studies

Test of Moderators (coefficient(s) 2): QM(df=1)=2.44, p=0.12

I^2(residual heterogeneity/unaccounted variability): 58.82%.

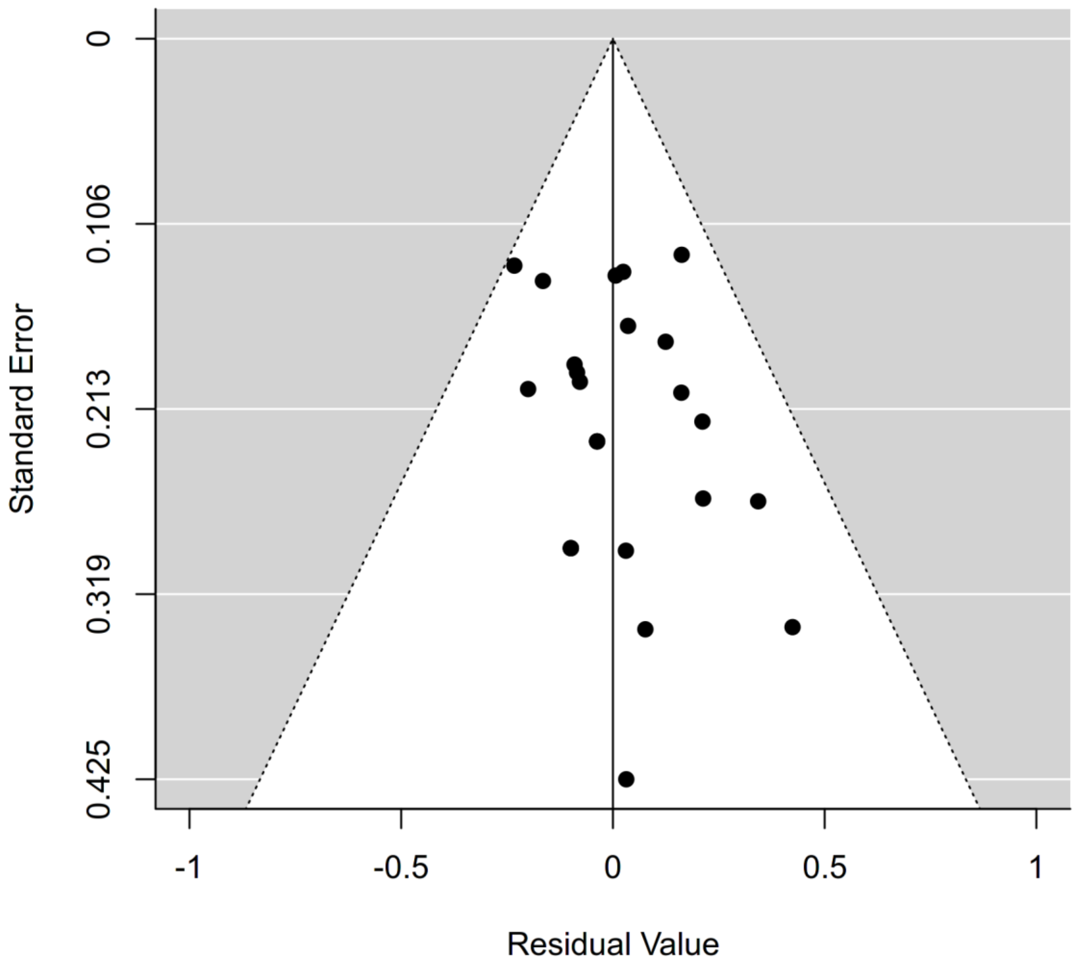


**Figure S7. Funnel-Plot for frequency of deceased patients showing residual value and standard error of included publications.** The analysis showed no remarkable publication bias.

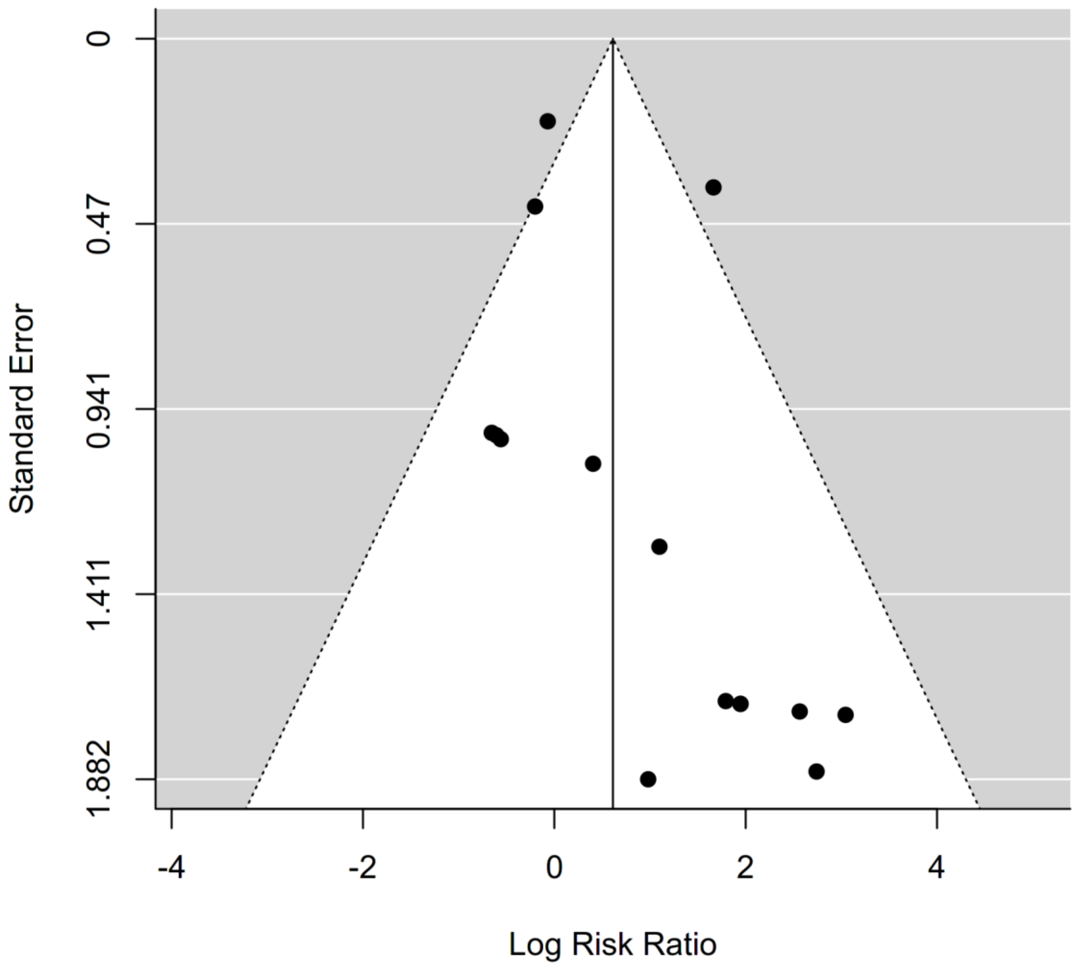


**Figure S8. Funnel Plot for effect of MT adherence and development of insidious onset MD in patients identified by NBS including non-informative studies showing residual value and standard error of included publications.** The analysis showed no remarkable publication bias.

*MD,* movement disorder; MT, maintenance treatment; *NBS,* newborn screening

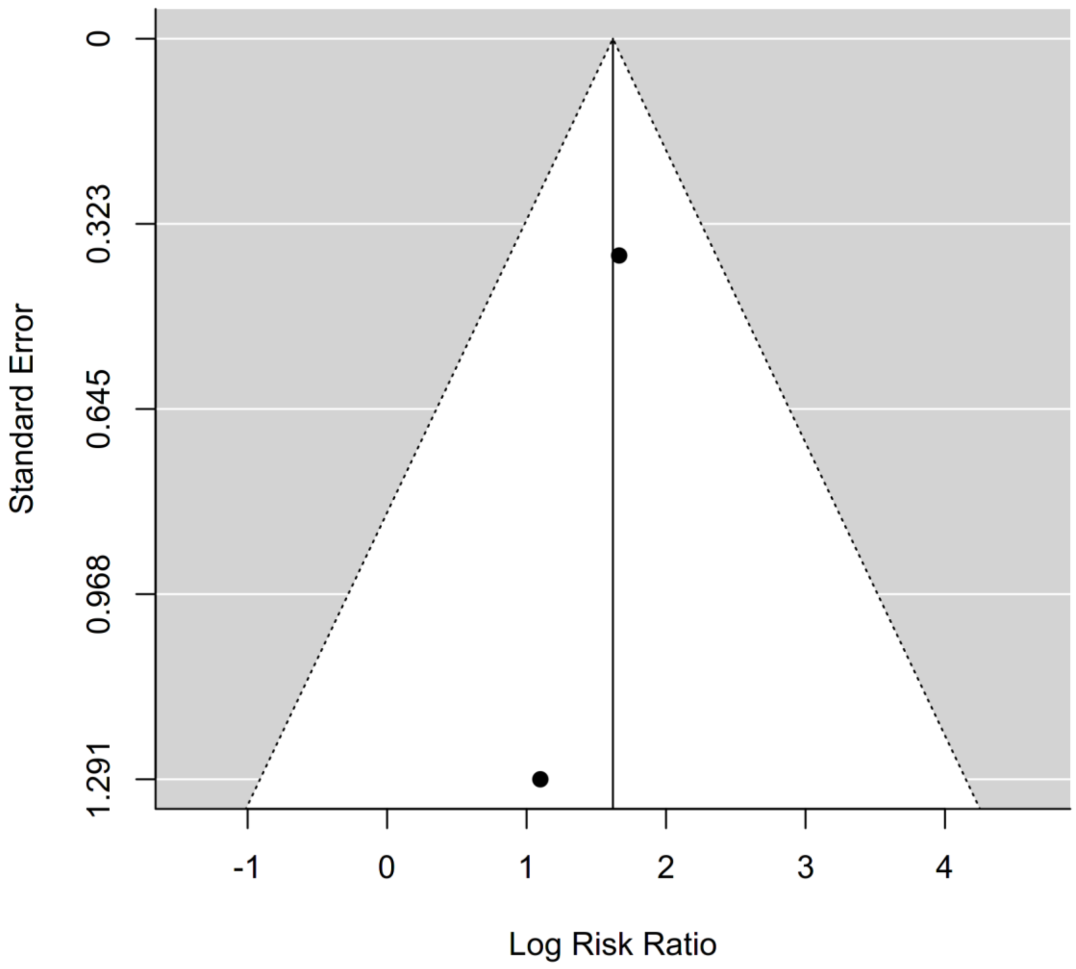


**Figure S9. Funnel Plot for effect of MT adherence and development of insidious onset MD in patients identified by NBS excluding non-informative studies showing residual value and standard error of included publications.** The analysis showed no remarkable publication bias.

*MD,* movement disorder; MT, maintenance treatment; *NBS,* newborn screening

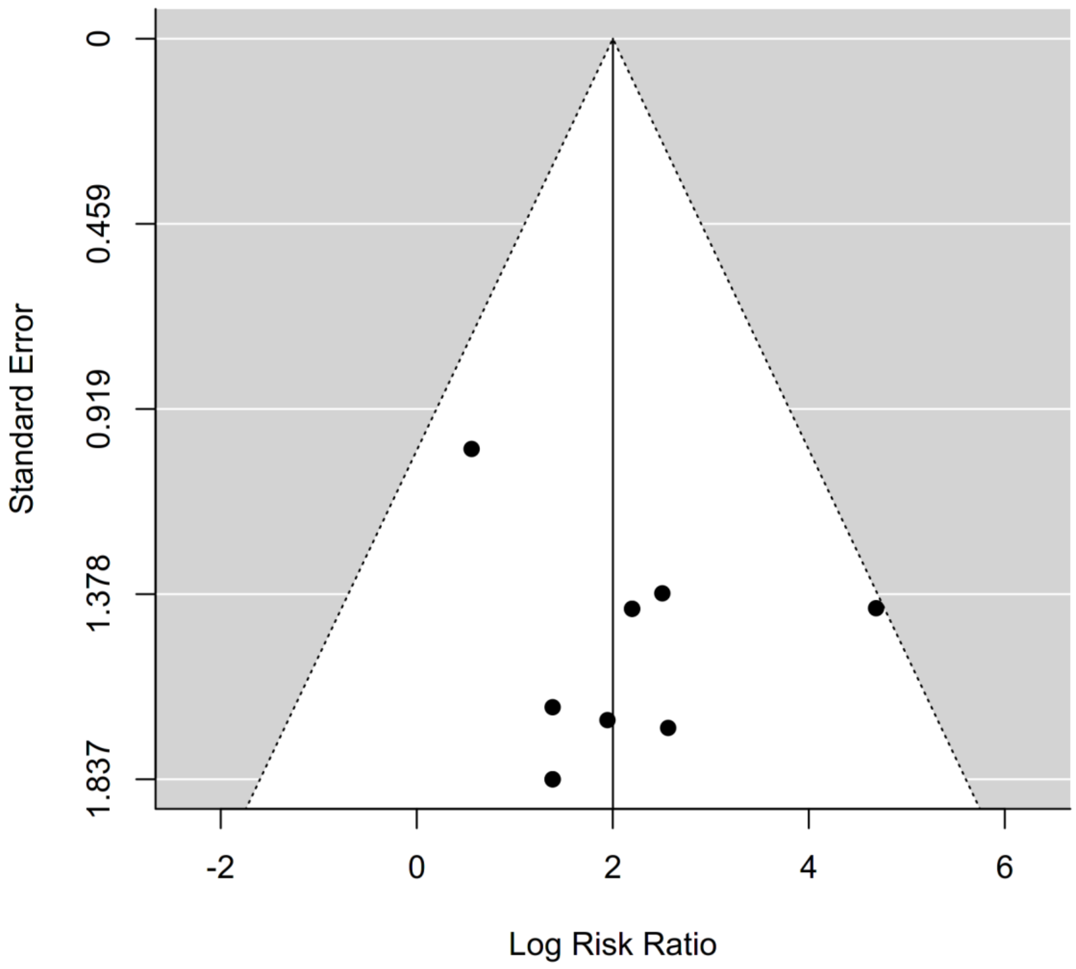


**Figure S10. Funnel Plot for effect of delayed ET on development of acute onset MD in patients identified by NBS including non-informative studies showing residual value and standard error of included publications.** The analysis showed no remarkable publication bias.

*ET,* emergency treatment; *MD,* movement disorder; *NBS,* newborn screening

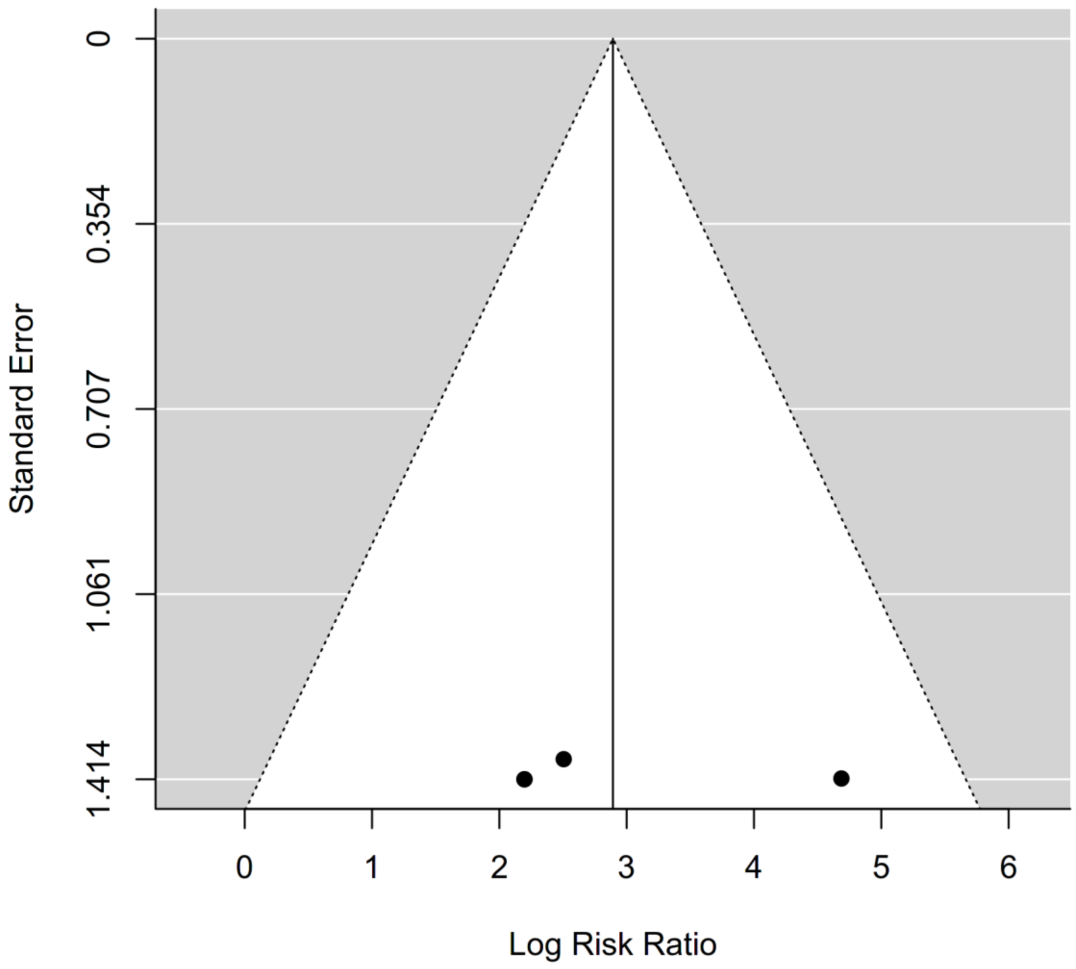


**Figure S11. Funnel Plot for effect of delayed ET on development of acute onset MD in patients identified by NBS excluding non-informative studies showing residual value and standard error of included publications.** The analysis showed no remarkable publication bias.

*ET,* emergency treatment; *MD,* movement disorder; *NBS,* newborn screening

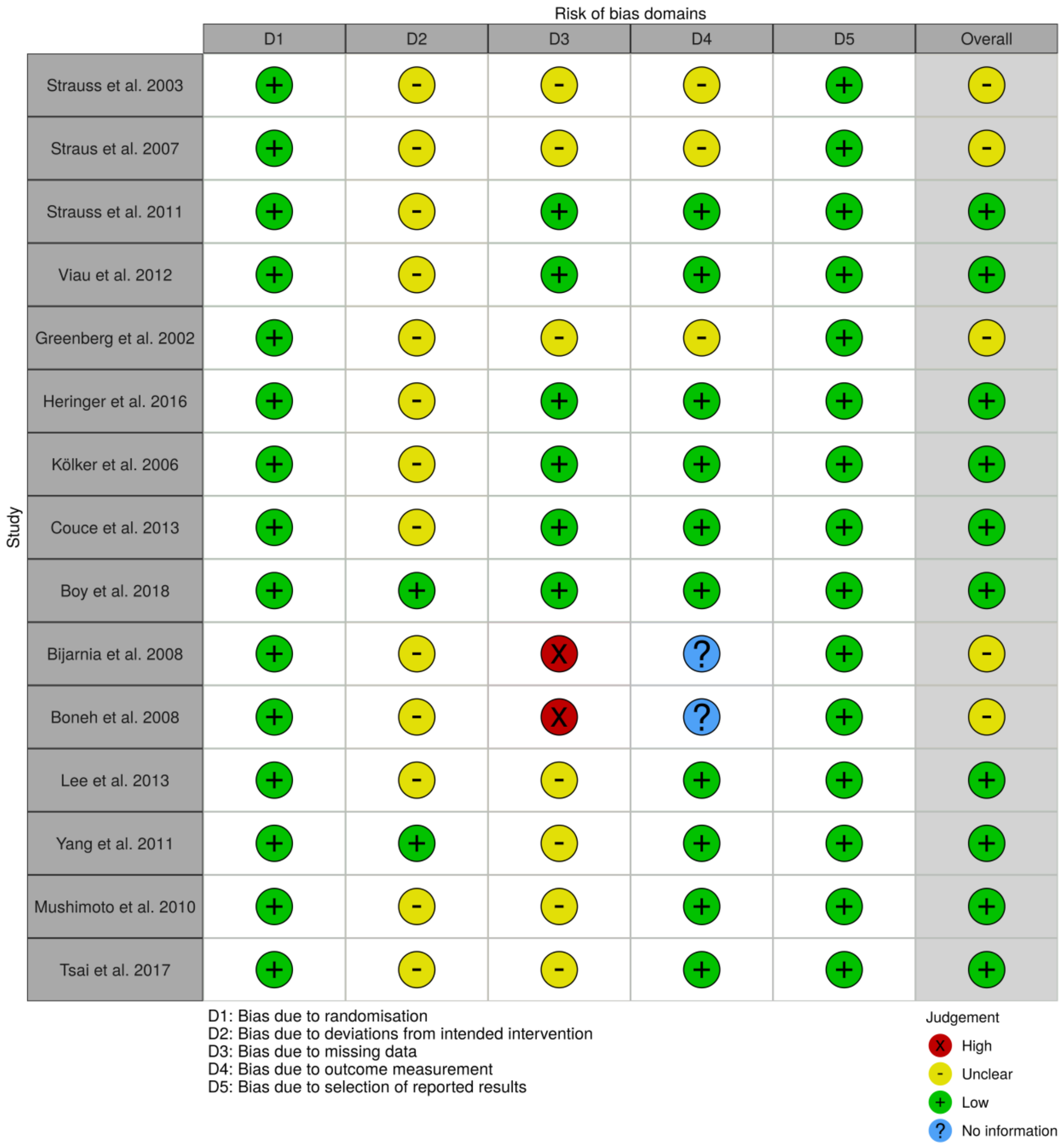


**Figure S12. Risk-of-bias assessment indicating the main five domains of risk-of-bias for each included study.** The method is recommended by the Cochrane Handbook (reference no. 34) and the Robvis risk-of bias tool (no. 35) which were used to create this figure.
